# An amino acid transporter subunit as an antibody–drug conjugate target in colorectal cancer

**DOI:** 10.1186/s13046-023-02784-0

**Published:** 2023-08-09

**Authors:** Juan Carlos Montero, Sofía del Carmen, Mar Abad, José M. Sayagués, Antonio Barbáchano, Asunción Fernández-Barral, Alberto Muñoz, Atanasio Pandiella

**Affiliations:** 1 Institute of Biomedical Research of Salamanca (IBSAL), Instituto de Biología Molecular y Celular del Cáncer (CSIC-Universidad de Salamanca), Salamanca, Spain; 2 Department of Pathology and IBSAL, University Hospital of Salamanca, Salamanca, Spain; 3 CIBERONC, Madrid, Spain; 4 Instituto de Investigaciones Biomédicas ‘Alberto Sols’, CSIC-Autonomous University of Madrid, and Instituto de Investigación Sanitaria del Hospital Universitario La Paz, Madrid, Spain

**Keywords:** CD98hc, Antibody–drug conjugates, Colorectal cancer, Targeted therapy

## Abstract

**Background:**

Advanced colorectal cancer (CRC) is difficult to treat. For that reason, the development of novel therapeutics is necessary. Here we describe a potentially actionable plasma membrane target, the amino acid transporter protein subunit CD98hc.

**Methods:**

Western blot and immunohistochemical analyses of CD98hc protein expression were carried out on paired normal and tumoral tissues from patients with CRC. Immunofluorescence and western studies were used to characterize the action of a DM1-based CD98hc-directed antibody–drug conjugate (ADC). MTT and Annexin V studies were performed to evaluate the effect of the anti-CD98hc-ADC on cell proliferation and apoptosis. CRISPR/Cas9 and shRNA were used to explore the specificity of the ADC. In vitro analyses of the antitumoral activity of the anti-CD98hc-ADC on 3D patient-derived normal as well as tumoral organoids were also carried out. Xenografted CRC cells and a PDX were used to analyze the antitumoral properties of the anti-CD98hc-ADC.

**Results:**

Genomic as well proteomic analyses of paired normal and tumoral samples showed that CD98hc expression was significantly higher in tumoral tissues as compared to levels of CD98hc present in the normal colonic tissue. In human CRC cell lines, an ADC that recognized the CD98hc ectodomain, reached the lysosomes and exerted potent antitumoral activity. The specificity of the CD98hc-directed ADC was demonstrated using CRC cells in which CD98hc was decreased by shRNA or deleted using CRISPR/Cas9. Studies in patient-derived organoids verified the antitumoral action of the anti-CD98hc-ADC, which largely spared normal tissue-derived colon organoids. In vivo studies using xenografted CRC cells or patient-derived xenografts confirmed the antitumoral activity of the anti-CD98hc-ADC.

**Conclusions:**

The studies herewith reported indicate that CD98hc may represent a novel ADC target that, upon well-designed clinical trials, could be used to increase the therapeutic armamentarium against CRC.

**Supplementary Information:**

The online version contains supplementary material available at 10.1186/s13046-023-02784-0.

## Background

Colorectal cancer (CRC) is the third most frequently diagnosed cancer, and accounts for 9.4% of all cancer deaths [1]. While therapeutic developments have improved, its prognosis in the metastatic setting is poor [2]. Therefore, more effective therapeutic strategies should be developed to improve the overall survival and quality of life of patients with this disease.

Monoclonal antibodies targeting cell surface proteins have shown clinical benefit in several oncological diseases, including CRC [3]. A sophisticated version of these antibodies are antibody–drug conjugates (ADCs). ADCs consist of three components: an antibody directed to a cell surface protein, a cytotoxic drug, and a chemical linker used to attach the cytotoxic to the antibody [4]. Whereas several ADCs have reached the oncology clinic, none of them has been approved for use in CRC [5]. Yet, because of their success in the treatment of other tumor types, some ongoing studies are analyzing the therapeutic value of ADCs that target different cell surface proteins in CRC. In this sense, ADCs against CEACAM5 (labetuzumab-govitecan) [6, 7], HER3 (patritumab-deruxtecan) [8, 9], or HER2 (trastuzumab-deruxtecan and trastuzumab-emtansine) [10] are currently being tested in CRC.

With the purpose of increasing the therapeutic options for different tumors, and considering the success of ADCs in the clinic, several studies have described different strategies to identify novel ADC targets [11–15]. These strategies are based on the analyses of cell surface proteins preferentially present in tumoral tissues as compared to non-tumoral tissues. That approach was recently used in triple negative breast cancer to elaborate a catalog of cell surface proteins differentially expressed in the tumor tissue [14], with the final objective of evaluating whether some of those proteins could be used with therapeutic purposes. One of the identified proteins was CD98hc, a cell surface glycoprotein that through heterodimerization with LAT1 participates in the uptake of essential amino acids [16, 17]. Several studies have shown that CD98hc is highly expressed in different tumors, and has been suggested to have a role in tumor progression [14, 16, 18–30] and metastatic dissemination [31]. Moreover, genetic evidence obtained in flies demonstrated that augmented expression of CD98hc favors tumor progression likely because concomitant up-regulation of several of the light chain transporter proteins that associate with CD98hc [32].

In CRC, a recent study evaluated the expression of CD98hc at the transcriptional and protein levels, showing that CD98hc is increased in carcinoma with respect to normal colonic mucosa and most benign CRC lesions [33]. This study also demonstrated that the expression of the proteins of the LAT1-CD98hc complex had no significant prognostic value in CRC patients. However, this later conclusion contrasts with the results of another report in which the levels of CD98hc were found to positively correlate with a worse prognosis in CRC [34].

The above commented works as well as others [35] suggest that CD98hc may represent a potential therapeutic target in CRC. Considering this, the necessity of novel therapeutics in CRC, and the successful antitumoral activity of an anti-CD98hc ADC in triple negative breast cancer [14], we decided to explore whether CD98hc could represent an actionable target in CRC. Using paired tumoral and non-tumoral samples from CRC patients, we here show that CD98hc is differentially expressed in normal versus tumoral tissue. Moreover, in vitro studies indicated that the CD98hc-ADC internalizes in CRC cell lines and exerts a potent antitumoral effect. Furthermore, experiments using patient-derived normal and tumoral CRC organoids, as well as studies using xenografted cell lines in mice and PDXs show that the anti-CD98hc ADC exerts ex vivo and in vivo antitumoral activity.

## Methods

### Reagents and antibodies

Dulbecco’s modified Eagle medium (DMEM), fetal bovine serum (FBS), penicillin, amphotericin B and streptomycin were purchased from Life Technologies (Carlsbad, CA, USA). 6-diamidino-2-phenylindole (DAPI) and 3-(4, 5-Dimethylthiazol-2-yl)-2, 5-Diphenyltetrazolium Bromide (MTT) were from Sigma-Aldrich (St Louis, MO, USA). Immobilon®-P transfer membranes were from Merck Millipore Corp. (Darmstadt, Germany). The Safe & Easy Toxin (SET™) was from Levena Biopharma (San Diego, CA, USA). Annexin V-FITC was from BD transduction Laboratories (San Jose, CA, USA). Other generic chemicals were from Sigma-Aldrich, USB Corporation (Cleveland, OH, USA) Roche Biochemicals (Hoffmann, Germany), or Merck (Darmstadt, Germany).

Antibodies against ERK1/2 (clone# C-9, reference sc-514302, WB: 1:5,000), calnexin (clone# E-10, reference sc-46669, WB: 1:10,000), cyclin B1 (clone# GNS1, reference sc-245, WB: 1:5,000), CD98hc (clone# 4F2, reference sc-59145 used for immunofluorescence, cell surface staining and to prepare the ADC, named in this paper as anti-CD98hc^ECTO^ or anti-CD98hc) and GAPDH (clone# H-12, reference sc-166574, WB: 1:10,000) were from Santa Cruz Biotechnology (Santa Cruz, CA, USA). Antibodies against CD98hc (clone# D3F9D, reference #47213 used in Western blot: 1:8,000 and immunohistochemistry experiments 1:500, termed anti-CD98hc^V509^), LAMP1 (clone# D2D11, reference #9091, IF: 1:200), phospho-HA2X (S139) (reference #2577, WB: 1:3,000), phospho-Rb (S780) (clone# C84F6, reference #3590, WB: 1:5,000), phospho-cdc2 (CDK1) (Y15) (reference #9111, WB: 1:8,000), PARP (reference #9542, WB: 1:6,000) and cleaved caspase 3 (clone# 5A1E, reference #9664, WB: 1:1,000) were from Cell Signaling Technologies (Beverly, MA, USA). Antibodies against cyclin A (clone# 25/Cyclin A, reference #611268, WB: 1:5,000), BUBR1 (clone# 9/BUBR1, reference 612503, WB: 1:3,500) and nucleoporin (reference N43620-150, IF: 1:200) were from BD transduction Laboratories. Antibody against CA2 (reference CSB-PA004370GA01HU, WB: 1:1,000), used in Western blot, was from Cusabio (Houston, TX, USA). The anti α -Tubulin (clone# DM1A, reference CP06, WB: 1:10,000) and anti phospho-H3 (S10) (reference #06–570, WB: 1:5,000) was from Merck Millipore (Darmstadt, Germany). The anti β-tubulin (clone# TUB 2.1, reference T4026, IF: 1:200) was from Sigma-Aldrich. The anti-DM1 antibody prepared against bovine serum albumin (BSA, from Sigma-Aldrich)-coupled DM1 (MedChemExpress, Sollentuna, Sweden) and the coupling of the anti-CD98hc to DM1 have been described [14]. Secondary HRP-conjugated antibodies recognizing mouse IgG and rabbit IgG were obtained from GE Healthcare Life Sciences (Piscataway, NJ, USA) (reference NA931, dilution 1:10,000) and Bio-Rad Laboratories (Hercules, CA, USA) (reference #1706515, dilution 1:20,000), respectively.

### Cell culture, cell proliferation and cell cycle assays

The cells were grown in Dulbecco's modified Eagle's medium (DMEM) (Caco-2, HCT116, HT29, SW480, SW620, and HaCaT) or in F-12K Medium (Kaighn´s Modification of Ham´s F-12 Medium) (LoVo) or in Leibovitz´s L-15 Medium (SW48) supplemented with 10% fetal bovine serum (FBS), containing high glucose (4500 mg/liter) and antibiotics (penicillin 100 U/ml, streptomycin 100 µg/ml). Cell lines were cultured at 37°C in a humidified atmosphere in the presence of 5% CO_2_ and 95% air. Cell authenticity was analyzed at the Hematology Service of the University Hospital of Salamanca. Human fibroblasts were obtained from normal tissue resected from patients undergoing surgery for CRCs. These cells were cultured in DMEM + 10% FBS.

Cell proliferation was assessed by MTT metabolization and cell cycle by propidium iodide staining, as previously described [36].

### Lentivirus production and infection

For lentivirus production [14], 4 µg of the following plasmids: pMDLg/RRE, pRSV-Rev and pMD2.G (Addgene, Cambridge, MA, USA), along with 8 µg of the pLKO.1 lentiviral plasmid containing a scramble shRNA (sh-Control) or the shRNAs for CD98hc (GE Dharmacon, Lafayette, CO, USA) were co-transfected by JetPEI® reagent (Polyplus-transfection, Illkirch, France) into HEK293T cells as described [37]. Twenty-four hours later, HEK293T medium was replaced with fresh medium and 48 h after the co-transfection, the medium containing lentiviral particles was collected, filtered, and used to infect HT29 and HCT116 cells after the addition of 6 µg/ml polybrene (Sigma-Aldrich). Cells were cultured for 48 h and were subsequently selected with 3 µg/ml puromycin (Sigma-Aldrich) for another 48 h. Five different shRNA sequences targeting CD98hc were tested (#3 GCCTGGACTCTTCTCCTATAT; #4 CGAGAAGAATGGTCTGGTGAA; #5 TCCGTGTCATTCTGGACCTTA; #6 GCTGGGTCCAATTCACAAGAA; #7 CTAGCTCATACCTGTCTGATT) and those that produced higher levels of knockdown (#3 and #7) were used.

### Immunoprecipitation and Western blotting

The preparation of cell extracts for protein analyses, immunoprecipitation and Western blotting procedures have been described [38, 39]. The preparation of the cell extracts from the paired samples (normal and tumor tissue) from patients with CRC were performed as described below in the xenograft studies. Depending on the molecular weight of the proteins to be analyzed, calnexin, tubulin, or GAPDH were used as loading controls. CA2 was used as a colon normal tissue control [40, 41]. Densitometric measurements of the bands were performed using the Image Lab™ Software Version 6.0.1 Bio-Rad Laboratories (Hercules, CA, USA), which was provided with a ChemiDoc apparatus. Stain free blot was performed by adding 50 µl of 2,2,2-Trichloroethanol to 10 ml of the SDS-PAGE gel solution. Detection of total protein was made in the ChemiDoc apparatus (Bio-Rad) following the manufacturer's instructions.

### Immunofluorescence microscopy

The immunofluorescence protocol for cell lines has been reported [42]. In short, cells were cultured on glass coverslips inserted into 35 mm dishes and treated with 10 nM of anti-CD98hc or anti-CD98hc-DM1. Cells on coverslips were washed with PBS/CM (1 mM CaCl_2_, 0.5 mM MgCl_2_ in PBS), fixed in 2% paraformaldehyde, and then washed with PBS/CM. Incubations were quenched with 50 mM NH_4_Cl. Cells were permeabilized (0.1% triton, 0.2% BSA) and then coverslips with cells incubated for 1 h in blocking solution, that contained PBS/CM with 0.2% BSA. The anti-LAMP1 (clone# D2D11, reference #9091, dilution 1:200), anti-β-tubulin (clone# TUB 2.1, reference T4026, dilution 1:200) or anti-nucleoporin (reference N43620-150, dilution 1:200) antibodies were added to the cells on the coverslips in blocking solution for 2 h at room temperature. After three washes for 10 min each in PBS with 0.2% BSA, the coverslips were incubated with Cy3-conjugated anti-mouse (dilution 1:500), Cy2-conjugated goat anti-rabbit (dilution 1:100) or Cy2-conjugated anti-mouse (dilution 1:100) antibodies in blocking solution for 30 min. Coverslips were washed three times for 10 min (in PBS with 0.2% BSA), and nuclei stained with DAPI before being mounted. Samples were analyzed by confocal immunofluorescence microscopy using a Leica TCS SP5 system (Leica Microsystems CMS, Wetzlar, Germany).

For immunofluorescence experiments in human organoids (20–40 µ m), they were seeded on 12-well plates (1,200 organoids/well). Two days later, they were incubated with anti-CD98hc antibody alone or conjugated with DM1 for the times indicated in the figure legends. After treatment, culture medium was removed, and organoids were washed in PBS. Fixation was performed in 3.7% paraformaldehyde for 60 min at room temperature. Organoids were then detached from the plate with a scrapper and collected in a 1.5 mL conical tube, washed in PBS and incubated in 0.1% glycine diluted in 1% PBS-BSA for 30 min at room temperature (RT). Organoids were then plated on microscope slides and permeabilized with 0.5% Triton-X-100 in PBS for 15 min and 0.01% Tween-20 in PBS for 5 min. Following three washes in 0.01% Tween-20 in PBS, organoids were incubated with secondary goat anti-mouse-Alexa 488 antibody (1:400) (Thermo Fisher Scientific, MA, USA) for 1 h at RT and nuclei were stained with DAPI. Images were taken using a DM2000 Leica microscope equipped with the LAS AF software (version 2.6.0.7266, Leica).

### FACS analyses

The procedure for the cell surface staining of CD98hc in HT29 cell and BT6224 tumors has been described [14]. Briefly, the tumor BT6224 was minced and treated with collagenase type 1 (Worthington) 1 mg/ml in Hanks Balanced Salt Solution (HBSS) for 2 h in agitation at 37°C. Later, the isolated cells were filtered with a cell strainer of 40 µm and washed twice with HBBS. Then, the cells were cultured in DMEM + 10% FBS for two days. Cells were treated with anti-CD98hc-DM1 (10 nM, 20 min at 37°C), trypsinized and collected in culture medium, centrifuged at 1200 r.p.m. for 5 min and resuspended in PBS + 2% BSA. Subsequently, cells were incubated with anti-Mouse FITC (1:100, Cytognos S.L., Salamanca, Spain) for 30 min in agitation at room temperature. Later, cells were washed twice with PBS + 2% BSA and the expression of CD98hc was analyzed by FACS using an Accuri C6 flow cytometer (BD transduction Laboratories).

For organoid cell surface staining of CD98hc, they were cultured for four days and then were extracted from the matrigel using dispase 1 mg/mL (Thermo Fisher Scientific) for 30 min. Subsequently, the organoids were incubated with TripLEx (diluted 1:4 in PBS) for 10 min and the cells were isolated by passing 10 times through a 21G syringe. Next, the cells were counted and incubated with anti-CD98hc-DM1 (10 nM, 30 min). Later, cells were washed twice with PBS + 0.1% BSA + EDTA 5 mM (wash buffer) and incubated with anti-Mouse Alexa 488 (1:400, Thermo Fisher Scientific, MA, USA) for 16 min. After two cell washes, the expression of CD98hc was analyzed by FACS (Cytoflex S cytometer, Beckman Coulter, Barcelona, Spain) using the Flow Jo (10.40.1) Software.

### Human samples

Fresh human tissues from CRC patients subjected to surgery were provided by the biobank of La Paz University Hospital-IdiPAZ (Madrid, Spain) or by PMC-BEOCyL (Comparative Molecular Pathology—Biobank Network of Oncological Diseases of Castilla y León, Salamanca, Spain), participating centres of the Spanish Biobank Network (www.redbiobancos.es). The study complied with all ethical regulations and was approved by the Ethics Committee of La Paz University Hospital (HULP-PI-1425 and HULP-PI-3169) and by the ethics committee of the University Hospital of Salamanca (Salamanca, Spain). All patients gave their informed consent prior to entering the study, according to the Declaration of Helsinki.

### Establishment of 3D normal and tumor organoid cultures

To establish human colon normal and tumor organoid cultures derived from colorectal cancer patients, normal mucosa and tumor biopsies were processed as previously described [40]. Biopsies were first incubated with a mixture of antibiotics (Primocin, gentamycin, fungizone) for 1 h at room temperature (RT).

To generate normal organoids cultures, samples were incubated with 10 mM dithiothreitol (DTT) for 10 min at RT and then with 8 mM EDTA solution for 60 min in slow rotation at 4C. Crypts were then isolated from the mucosa by shaking and embedded in Matrigel (Corning, MA, USA) that was seeded in drops on pre-warmed culture dishes. Following Matrigel solidification, complete “Normal” culture medium was added: 50% Advanced DMEM/F12, 50% Wnt3a-conditioned medium, 10 mM HEPES, 10 mM Glutamax, 10 mM nicotinamide (Sigma-Aldrich), 1 × N2, 1 × B27 (Thermo Fisher Scientific, MA, USA), 1 mM N-acetyl-L-cysteine (Sigma-Aldrich), 1:500 Primocin, 0.1 μg/mL Noggin (Peprotech, New Jersey, NJ, USA), 1 μg/mL gastrin (Tocris, Bristol, UK), 1 μg/mL RSPO1 (Sinobiological, Beijing, China), 50 ng/mL EGF (Peprotech), 0.02 μM PGE2 (Sigma-Aldrich), 1 μM LY-2157299 (Axon-Medchem, Groningen, The Netherlands), and 10 μM SB-202190 (Sigma-Aldrich).

Human tumor organoid cultures were generated as follows. Tumor biopsies were cut into small pieces and digested enzymatically in a suspension of 1 mg/mL collagenase type IV (Sigma-Aldrich) in PBS for 30 min at 37ºC with continuous shaking in a waterbath. To obtain single cells, we forced cell disaggregation by passing the suspension through an 18G syringe and single cells were collected after passing the solution through a 100-μm mesh filter. Finally, cells embedded in Matrigel were seeded on pre-warmed culture dishes until solidification and “Tumor” culture medium (“Normal” culture medium minus Wnt3a-conditioned medium, nicotinamide and RSPO1) was added.

For passaging, Matrigel was removed from organoids after incubation with dispase 1mg/mL (Thermo Fisher Scientific) for 30 min. Organoids were incubated with TripLEx (diluted 1:4 in PBS) for 5 min and passed through a 21G syringe. After washing the pelleted organoids in washing buffer (Advanced DMEM/F12, 10 mM HEPES, 10 mM Glutamax), disrupted organoids were embedded in Matrigel and seeded on pre-warmed culture dishes.

Drug activity in tumor organoids was performed following a previously published protocol [43]. Organoids were treated with 10 nM anti-CD98hc-DM1 or vehicle for 4 days, and cell viability was measured using the Cell Titer-Glo 3D Cell Viability kit assay (Promega, Madison, WI, USA).

### Xenograft studies

Mice were handled at the institute’s animal facility, and all treatments were done in accordance with the legal and institutional guidelines. Female BALB/c nude mice, 7 weeks old, were obtained from Charles River Laboratories (Wilmington, MA, USA). A total of 5 × 10^6^ HT29 cells in 50 µl of DMEM + 10% FBS and 50 µl of Matrigel (Corning) were injected subcutaneously into the right flank of the mice. When tumors reached 150 mm^3^, animals were randomized into two groups (*n* = 4–6 animals for each condition), and treatments were initiated by weekly intraperitoneal injection with 15 mg/kg of anti-CD98hc-DM1 or anti-CD98hc (three doses in total), or DM1 (0.14 mg/Kg, once per week, three doses in total). Tumor diameters were serially measured with a digital caliper (Proinsa, Vitoria, Spain) every 3–4 days (twice a week), and tumor volumes were calculated using the following formula: V = (L × W^2^)/2, where V = volume (cubic millimeters), L = length (millimeters), and W = width (millimeters). Tumor’s samples were obtained on day 21 after initiation of treatments after sacrificing the animals by CO_2_ inhalation, and immediately one piece was frozen in liquid nitrogen. Tumors that were frozen in liquid nitrogen were minced, washed with PBS, and homogenized (Dispomix, L&M Biotech, Holly Springs, NC, USA) in ice-cold lysis buffer (2 ml/100 mg of tumor). Homogenates were centrifuged at 10,000 × *g* for 20 min at 4°C, and the supernatants were transferred to new tubes.

### Colorectal cancer Patient Derived Xenograft (PDX) studies

Bioethical procedures were approved by the Ethics Committees of the University Hospital of Salamanca (patient-derived material) and the Ethics Committee of the Salamanca University (animal manipulation). Freshly resected tumor from patients with colorectal cancer were collected after surgery. The tumors were placed into DMEM medium supplemented with antibiotics (penicillin and streptomycin), antifungi (amphotericin B) and 10% FBS at RT. One-three hours later, the tumor tissues were cut into pieces of 3 × 3x3 mm and placed subcutaneously on the back into the left flank of 7 weeks old female BALB/c nude mice. When the tumors reached 600–1200 mm^3^ (P1: first generation of xenografts), the tumor was expanded to another mouse (P2). Again, when tumors reached 600–1200 mm^3^, the mice were sacrificed and then the tumor was expanded again to 8 mice. When tumors reached 230 mm^3^, the mice were separated in two groups (*n* = 4, for control or treated mice) and then treatment was started in the treatment group (q. 10 days by intraperitoneal injection) with 15 mg/kg of anti-CD98hc-DM1 (in total three doses). Tumor diameters were serially measured with a digital caliper (Proinsa, Vitoria, Spain) every 3–4 days (twice a week), and tumor volumes were calculated using the following formula: V = (L × W^2^)/2, where V = volume (cubic millimeters), L = length (millimeters), and W = width (millimeters). Tumoral samples were obtained on day 28 after initiation of treatments after killing the animals by CO_2_ inhalation, and immediately one piece was frozen in liquid nitrogen. Protein extraction from tumors was performed as in Xenograft studies.

### Tissue array and immunohistochemical analyses of CD98hc

Colon cancer tissue samples were formalin fixed and paraffin embedded. Three-micrometer sections were cut with a microtome (Leica Microsystems GmbH, Wetzlar, Germany) and transferred to adhesive-coated slides. Immunohistochemistry was carried out on these sections using a Leica BOND-III Fully Automated IHC and ISH Staining System (Leica Microsistemas S.L.U. All Microscopy and Histology, Barcelona, Spain) following the manufacturer's instructions. CD98hc expression was analyzed using the anti-CD98hc (Cell Signaling Technology, clone# D3F9D, reference #47,213) at a 1:500 dilution during 20 min.

The tissue microarray (TMA) used to analyze levels of CD98hc in 26 primary tumors, 34 liver metastases and 16 lymph node metastases by Immunohistochemical was created as described [44]. The CD98hc score in the TMA was calculated as follows: i) % of neoplastic cells stained: 0- 0; 1- < 10%; 2- 10–50%; 3- 50–75%; 4- > 75%. ii) Intensity: 0 negative; (1) + weak; (2) +  + moderate; (3) +  +  + intense.

### Deletion of CD98hc by CRISPR/Cas9

HT29 cells (plate of 35 mm, 50–80% confluence) were transfected with 1.5 µg of a mixture of 3 plasmids targeting 3 different sequences of CD98hc (CD98 CRISPR/Cas9 KO, ref. SC-400501) each encoding the Cas9 nuclease and a target-specific 20 nt guide RNA (gRNA) designed for maximum knockout efficiency of CD98 and 1.5 µg CD98 HDR plasmid consisting of a pool of 2–3 plasmids, each containing a homology-directed DNA repair (HDR) template corresponding to the cut sites generated by the CD98 CRISPR/Cas9 KO Plasmid and that allows the selection of cells containing a DSB induced by CD98 CRISPR/Cas9 KO Plasmid) (Santa Cruz Biotechnology, sc-400501) using JetPEI DNA transfection reagent. Three days later, the cells were selected with 3 µg/ml of puromycin for two weeks (medium was renewed every 3 or 4 days). Cells were maintained for four additional weeks without puromycin and when clones were visible, they were isolated. Individual clones were grown in 24-multi-well plates and subsequently expanded. The expression of CD98hc of the different clones was analyzed by western blot.

### Statistical analyses and in silico studies

Statistical analyses were performed using the software package SPSS 15.0 (SPSS Inc. Chicago, IL, USA). Comparison of continuous variables between two groups for in vitro assays were performed using a two-sided Student’s *t*-test, unless otherwise indicated. Differences were considered statistically significant when *p* < 0.05. All experiments were repeated at least twice. Representative results of the findings are shown.

In silico evaluation of the expression of CD98hc in human samples was carried out using the TNMplot and GENT2 online databases. Expression units are automatically given in those databases. Statistically significant differences between normal and tumoral samples are marked in red in the data obtained from the TNMplot database.

## Results

### Expression of CD98hc in colon cancer

**Fig. 1:**
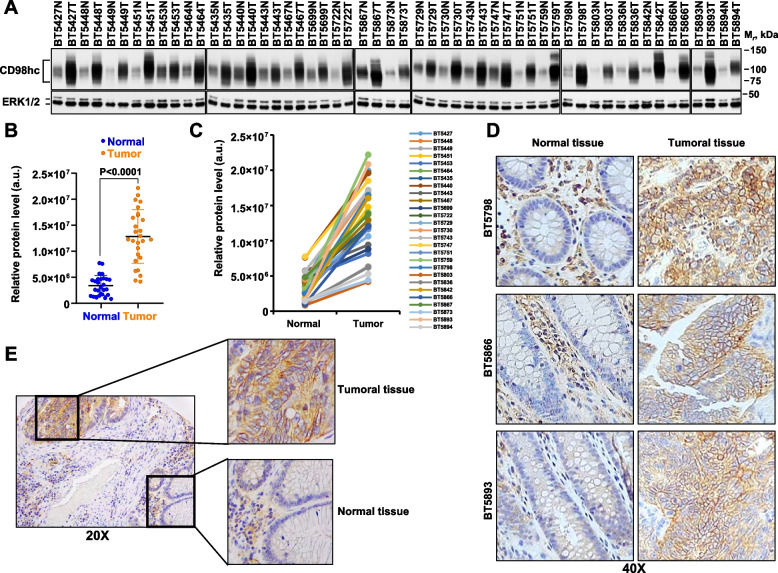
Expression of CD98hc in normal and tumoral tissues from patients with CRC. **A** Expression of CD98hc in paired samples (normal and tumoral colon tissue) of patients with CRC. CD98hc was analyzed by Western using the anti-CD98hc^V509^ antibody. ERK1/2 was used as a loading control. *N* = normal tissue, T = tumoral tissue. The position of the molecular weight markers is shown at the right. **B** and **C** represent the relative protein levels in arbitrary units of CD98hc of the data shown in A. *P* value in B was calculated using Mann Whitney U test. Quantitation of CD98hc was made as described in the methods section. **D** Immunohistochemical staining of CD98hc in paired colon samples (normal and tumoral tissue) stained with the anti-CD98hc^V509^ antibody. Magnification: 40X. **E** Immunohistochemical staining of CD98hc in a sample of a patient which includes tumoral as well as normal tissue. Magnification: 20 X

Postoperative assessment of CD98hc expression was performed in paired normal and tumoral tissue samples from 27 patients. Western blot analyses with an antibody that recognizes an epitope around Val509, located in the extracellular region of CD98hc (anti-CD98hc^V509^), showed expression of CD98hc in both normal as well as tumoral CRC tissue samples (Fig. 1A). The expression of CD98hc was significantly (*p* < 0.0001) higher in the tumoral samples when compared to their normal colonic counterparts, in all the 27 cases analyzed (Fig. 1A, B and C). These data were in concordance with those obtained using the TNMplot online tool [45] for 41 paired samples (accessed December 2022, supplementary Fig. 1A). Additional in silico studies using the Gepia2 online tool, that allows comparison of expression between normal and tumoral tissues [46], showed that CD98hc (gene name *SLC3A2*) expression was significantly (*p* = 0.01) higher in tumoral than in normal colonic tissue (supplementary Fig. 1B). Differences in the expression of CD98hc between non-paired normal tissue and tumoral CRC were also found using TNMplot (supplementary Fig. 1C). Analyses of online available data indicated that CD98hc expression was frequently up-regulated in tumoral tissues when compared to normal ones (supplementary figure 1G and H).

Immunohistochemical studies using the anti-CD98hc^V509^ antibody showed that the staining of CD98hc in the tumoral tissue of paired samples was substantially higher than the staining present in the normal tissue (Fig. 1D). The CD98hc staining pattern in the tumoral tissues was typical of a cell surface protein (membranous staining). In the normal paired samples, CD98hc staining was mainly restricted to the inflammatory stromal component of lamina propria of the intestinal mucosa. In samples that included normal as well as tumoral tissue, the staining of CD98hc in normal colon was substantially lower than the staining present in the adjacent tumoral areas (Fig. 1E). Metastatic tissues (lymph nodes or liver metastases) retained expression of CD98hc (supplementary Fig. 1D-F).

### Antibody-mediated internalization of CD98hc in colon cancer cells

To explore the potential antitumoral effect of CD98hc targeting in CRC, we followed a strategy previously shown to be successful in triple negative breast cancer [14]. This strategy is based on the construction of an ADC, using an antibody that recognizes the ectodomain of CD98 (anti-CD98hc^ECTO^), coupled to the potent antimicrotubular agent DM1. Of note, the antibody used for the western blot and immunohistochemical studies, the anti-CD98hc^V509^ antibody, cannot be used for this purpose as it does not recognize native CD98hc.

**Fig. 2:**
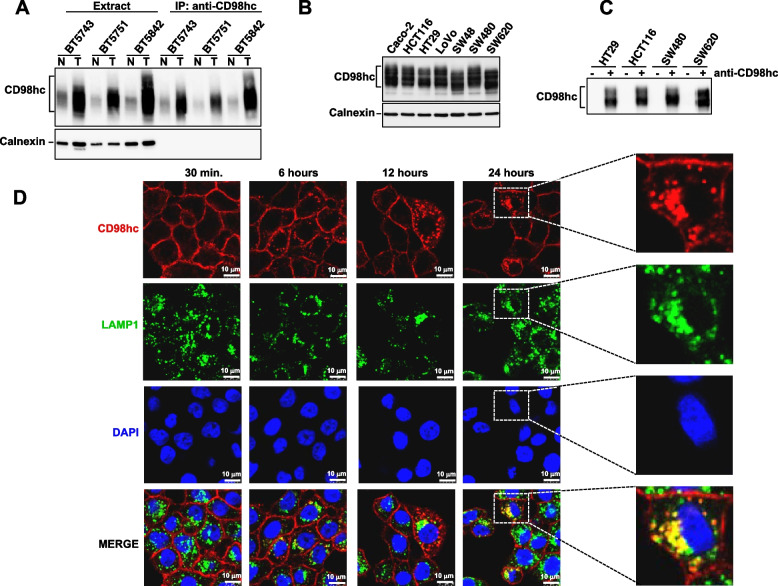
Internalization of an antibody against CD98hc in colon cancer cells. **A** Interaction of the anti-CD98hc^ECTO^ with native cell surface CD98hc. One mg of tissue extract from three paired patient samples (normal and tumoral) was immunoprecipitated with the anti-CD98hc^ECTO^ antibody and the immunocomplexes and extracts of these samples were analyzed by Western blot with the anti-CD98hc^V509^ antibody. Cell extracts (50 µg) from the same samples were also loaded. Calnexin was used as a loading control for the cell extract samples. **B** Expression of CD98hc in a panel of colon cancer cell lines. Colon cancer cell lines were lysed, and cell extracts (20 µg) used to identify CD98hc by Western blot with the anti-CD98hc^V509^ antibody. Calnexin was used as a loading control. **C** Cell surface immunoprecipitation of CD98hc. Four CRC cell lines were in vivo treated or not with 10 nM of anti-CD98hc^ECTO^ for 30 min at 37°C. Cells were lysed and cell extracts precipitated with protein A-sepharose. CD98hc in those immunoprecipitates was analyzed by Western blot with the anti-CD98hc^V509^ antibody. **D** Internalization of the anti-CD98hc^ECTO^ in HT29 cells, analyzed by immunofluorescence. The cells were seeded on coverslips and treated with 10 nM of anti-CD98hc^ECTO^ for 30 min, 6, 12 and 24 h. The images at the right correspond to magnifications of a cell present in the images obtained at 24 h. The colocalization of CD98hc and LAPM1 is show in the merged images

Initial experiments were designed to verify whether the anti-CD98hc^ECTO^ antibody interacted with native cell surface CD98hc and internalized inside colon carcinoma cells. The anti-CD98hc^ECTO^ antibody immunoprecipitated CD98hc from patient-derived samples (Fig. 2A), suggesting that it recognizes undenatured CD98hc. To explore this further, we then turned into CRC cell lines, in which Western studies demonstrated expression of CD98hc (Fig. 2B). Cell surface immunoprecipitation experiments using anti-CD98hc^ECTO^ showed that this antibody was able to interact with cell surface and native CD98hc (Fig. 2C).

Immunofluorescence studies were carried out to explore the potential internalization dynamics of the anti-CD98hc^ECTO^ antibody in CRC cells. To this end, HT29 or SW480 cells were incubated for different times with a saturating dose (10 nM) of the CD98hc^ECTO^ antibody. After 30 min of incubation, the anti-CD98hc^ECTO^ antibody stained the cell surface (Fig. 2D and supplementary Fig. 2A). Upon 24 h of incubation, in addition to the membrane staining, intracellular accumulation of the antibody following a dotted pattern was observed. Colocalization studies showed that this punctate staining was coincident with the lysosomal marker LAMP1.

### Antiproliferative activity of an anti-CD98hc ADC in colon cancer cells

Since the above data confirmed that the CD98hc^ECTO^ antibody was able to bind cell surface CD98hc and internalized to the lysosomes of CRC cells, we decided to use it as a skeleton to construct an anti-CD98hc ADC, using as a payload the antimicrotubular agent DM1. To check that DM1 efficiently coupled to the CD98hc^ECTO^ antibody, western blotting analyses of the uncoupled and coupled antibody were carried out using an anti-DM1 antibody [14]. As controls, we used commercially available trastuzumab as well as trastuzumab-DM1 (T-DM1). While the anti-DM1 antibody recognized the coupled DM1 in both T-DM1 and anti-CD98hc^ECTO^-DM1, it failed to recognize nude, uncoupled trastuzumab or anti-CD98hc^ECTO^ (supplementary Fig. 2B). Moreover, the intensity of the signal obtained in the Ig heavy chain of anti-CD98hc^ECTO^ was very similar to that obtained from the commercially available T-DM1. These experiments demonstrated that DM1 efficiently coupled to the anti-CD98hc^ECTO^ antibody. Moreover, FACS analyses demonstrated that the anti-CD98hc^ECTO^-DM1 antibody (hereon referred to as anti-CD98hc-DM1) retained capability to interact with surface-exposed CD98hc present in intact (i) HT29 cells (supplementary Fig. 2C), (ii) cells derived from a PDX grown from a patient with CRC (supplementary Fig. 2D), (iii) cells isolated from a tumoral organoid derived from a patient with CRC (supplementary Fig. 2E).

**Fig. 3:**
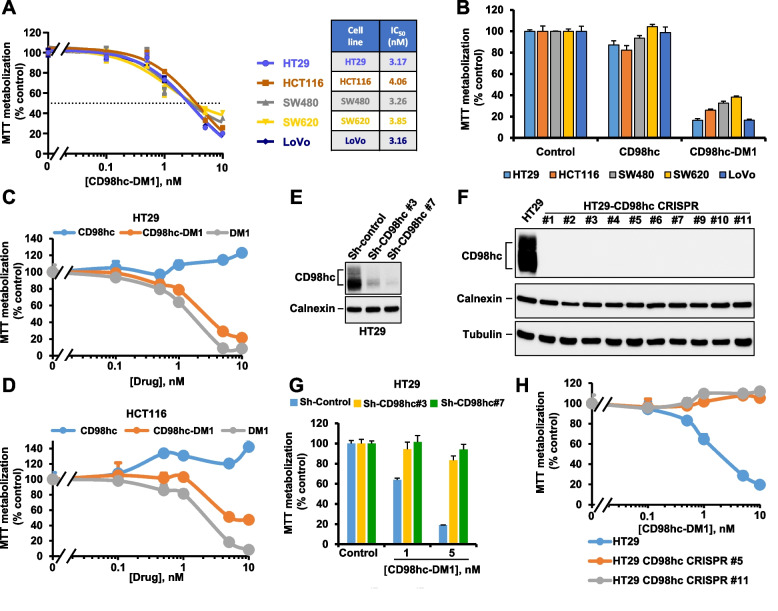
Anti-proliferative activity of the anti-CD98hc ADC in colon cancer cell lines. **A** Dose–response analyses of anti-CD98hc-DM1 in a panel of CRC cell lines. Cells were treated with the ADC for four days at the indicated doses. The data are plotted as the percentage of MTT metabolization with respect to control. Results are shown as the mean ± SD of quadruplicates of an experiment repeated two times. The table on the right indicates the IC_50_ (nM) of anti-CD98hc-DM1 for each cell line. **B** Effect of nude anti-CD98hc and anti-CD98hc-DM1 on the proliferation of CRC cell lines. Cells were treated with anti-CD98hc or anti-CD98hc-DM1 10 nM for four days. The data are plotted as the percentage of MTT metabolization with respect to control. Results are shown as the mean ± SD of triplicates of an experiment repeated twice. **C** and **D** HT29 (**C**) and HCT116 (**D**) cells were treated with the doses indicated of anti-CD98hc, anti-CD98hc-DM1 or DM1 for four days. The data are plotted as the percentage of MTT metabolization with respect to control. Results are shown as the mean ± SD of triplicates of an experiment repeated twice. **E** HT29 cells were infected with lentivirus containing the shRNA control (sh-Control) or the shRNA sequences targeting CD98hc (sh-CD98hc #3 and sh-CD98hc #7). Knockdown efficiency was verified by Western with the anti-CD98hc^V509^ antibody. Calnexin was used as a loading control. **F** Knockout of CD98hc in HT29 cells by CRISPR/Cas9. Parental HT29 cells and ten different clones knocked out for CD98hc were lysed and the levels of expression of CD98hc analyzed by Western blot with the anti-CD98hc^V509^ antibody. Calnexin and tubulin were used as a loading controls. **G** Impact of CD98hc knockdown on the antiproliferative effect of anti-CD98hc-DM1. HT29 cells were treated with anti-CD98hc-DM1 (1 nM and 5 nM) for four days. The data are plotted as the percentage of MTT metabolization with respect to control. Results are shown as the mean ± SD of triplicates of an experiment repeated twice. **H** Dose–response analyses of the effect of anti-CD98hc-DM1 in the proliferation of parental and CD98hc CRISPR #5 and #11 HT29 cells. Results are shown as the mean ± SD of quadruplicates of an experiment repeated three times

To explore the antiproliferative effect of anti-CD98hc-DM1, five CRC cell lines were treated with different doses of the anti-CD98hc-DM1 ADC for four days. These studies showed a dose-dependent decrease in MTT metabolization, used as a surrogate measurement of cell number, in the five CRC cell lines studied (Fig. 3A). The IC_50_ values were similar among the different cell lines analyzed, ranging from 3.16 to 4.06 nM. In contrast, treatment with the nude anti-CD98hc^ECTO^ antibody (hereon referred to as anti-CD98hc) did not substantially affect the proliferation of any of the cell lines tested (Fig. 3B), suggesting that the payload coupled to the antibody is the main responsible for the decrease in cell proliferation. Dose–response studies also showed that HT29 and HCT116 cells responded similarly to anti-CD98hc-DM1 and to the free payload DM1 (Fig. 3C and D). In agreement with the data shown in Fig. 3B, these dose–response experiments also confirmed that the nude antibody did not affect the proliferation of HT29 and HCT116 cells at any of the concentrations tested.

To explore the specificity of the antiproliferative action of the anti-CD98hc-DM1 ADC, CD98hc was decreased by shRNAi (Fig. 3E). In addition, CRISPR/Cas9 was used to generate several CD98hc-null HT29 cell clones (Fig. 3F). While in the parental HT29 cell line infected with a control shRNAi the ADC exerted an antiproliferative effect at both 1 nM and 5 nM, HT29 cells with lower levels of CD98hc were less sensitive to the action of the anti-CD98hc-DM1 (Fig. 3G). Analogous results were obtained when the antiproliferative effect of anti-CD98hc-DM1 was analyzed in two clones (HT29 CD98hc CRISPR#5 and #11) in which CD98hc expression was eliminated by means of CRISPR/Cas9 (Fig. 3H). Of note, MTT metabolization studies showed that the HT29 CD98hc CRISPR#5 grew similarly to the parental cells (Supplementary Fig. 3A).

The action of the anti-CD98hc-DM1 ADC was also explored in non-tumoral human fibroblasts obtained from normal colon, as well as in immortalized HaCaT keratinocytes. The latter expressed levels of CD98hc similar to those of the CRC cell lines, while the human fibroblast expressed much less (supplementary Fig. 3B). As shown in supplementary Fig. 3C, the anti-CD98hc-DM1 ADC exerted an antiproliferative effect on HaCaT similar to that observed in HT29 cells. In contrast, the anti-CD98hc-DM1 ADC had a very marginal effect on the proliferation of normal fibroblasts.

### The CD98hc-ADC induces cell cycle arrest in mitosis and mitotic catastrophe

**Fig. 4:**
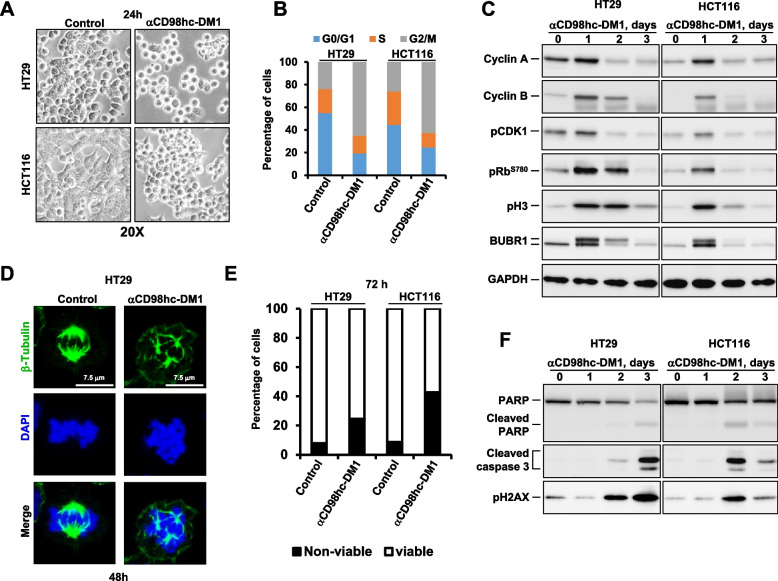
The anti-CD98hc-DM1 antibody induces cell cycle arrest in mitosis and mitotic catastrophe. **A** Effect of anti-CD98hc-DM1 (10 nM, 24 h) on the morphology of HT29 and HCT116 cells grown as monolayers. **B** Effect of anti-CD98hc-DM1 (10 nM, 24 h) on the distribution of the different cell cycle phases (G0/G1, S and G2/M) in HT29 and HCT116 cell lines. **C** Effect of anti-CD98hc-DM1 on the levels of expression and phosphorylation of proteins implicated in cell cycle progression. HT29 and HCT116 cells were treated with anti-CD98hc-DM1 (10 nM) and lysed at the indicated times. The levels of expression or phosphorylation of the different proteins studied were analyzed by Western. GAPDH was used as loading control. **D** Action of anti-CD98hc-DM1 on spindle assembly and organization. HT29 cells seeded on coverslips were treated with CD98hc-DM1 (10 nM) for 48 h, fixed and stained. β-Tubulin in green and DAPI in blue. Scale bars = 7.5 µm. **E** Percentage of viable (Annexin V-negative/PI-negative) and non-viable HT29 and HCT116 cells after 72 h of treatment with 10 nM anti-CD98hc-DM1. **F** Effect of anti-CD98hc-DM1 on the levels of different apoptosis-related proteins

The mechanism of the antiproliferative action of the anti-CD98hc-DM1 ADC was then analyzed in CRC cell lines. Visual inspection of HT29 or HCT116 cells treated with the ADC showed that the drug caused rounding of the cells after 24 h of treatment (Fig. 4A). Flow cytometry experiments indicated that the anti-CD98hc-DM1 caused accumulation of cells in G2/M cell cycle phases and caused a concomitant decrease in G0/G1 either at 24 (Fig. 4B) or 48 h (supplementary Fig. 3D) of treatment. Western blotting analyses showed that the ADC caused accumulation of mitotic markers pHistone-H3 and pBUBR1 after 1 day of treatment (Fig. 4C). An increase in cyclin A and cyclin B were also observed. Moreover, in both cell lines an increase was observed in Rb phosphorylation at S780, which is consistent with a state of hyperphosphorylation of Rb until late mitosis (Fig. 4C) [47, 48].

Immunofluorescence experiments showed that the anti-CD98hc-DM1 ADC induced aberrant mitotic spindles at 48 h of treatment (Fig. 4D and supplementary Fig. 3E). Moreover, we observed giant multinucleated cells in HT29 and HCT116 cells treated with anti-CD98hc-DM1 for 72 h (supplementary Fig. 3F). The presence of these aberrant mitotic spindles was indicative of mitotic catastrophe, a form of cell death triggered by deficient mitotic progression [49, 50]. In line with this, staining with Annexin V/propidium iodide showed that the anti-CD98hc-DM1 ADC was able to provoke a substantial increase in non-viable cells at 72 h posttreatment (Fig. 4E). Accordingly, treatment of HT29 and HCT116 cells for 2–3 days with anti-CD98hc-DM1 provoked an increase in cleaved forms of caspase 3 and PARP (Fig. 4F). A parallel increase in the levels of the DNA damage marker pH2AX was also detected. All these data indicate that treatment of colon carcinoma cell lines with the anti-CD98hc-DM1 ADC caused cell cycle arrest which progresses into cell death.

### Action of the CD98hc-DM1 ADC on normal and tumoral patient-derived organoids

**Fig. 5:**
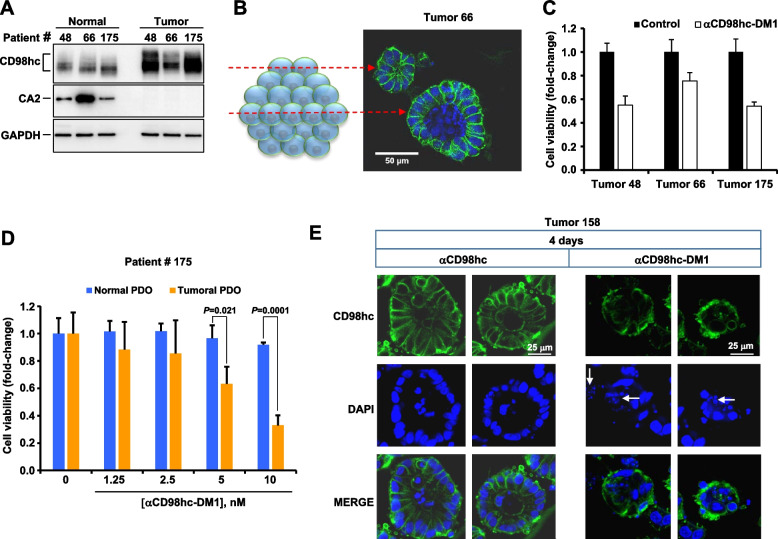
The anti-CD98hc-DM1 has anti-proliferative effect on tumoral patient-derived organoids. **A** Levels of expression of CD98hc in normal and tumoral PDOs. Levels of expression of CD98hc and CA2 (normal colon marker) were analyzed by Western blot with the anti-CD98hc^V509^ antibody. GAPDH was used as a loading control. **B** Immunofluorescence detection of CD98hc in tumoral PDOs. Tumoral PDOs were incubated with 10 nM of the anti-CD98hc^ECTO^ antibody for 1.5 h and the subcellular distribution of CD98hc was analyzed by immunofluorescence. Scale bar = 50 µm. The drawing on the left represents an organoid and the red dashed lines indicate the organoid regions where images were taken. **C** Evaluation of anti-CD98hc ADC on the cell viability of different tumoral PDOs. The tumoral PDOs were treated with 10 nM of anti-CD98hc-DM1 for 4 days and the cell viability was analyzed as indicated in the methods sections. Results are shown as the fold-change respect to untreated organoids ± SD of triplicates of an experiment repeated twice. **D** Dose–response analyses of the effect of anti-CD98hc-DM1 on cell viability of paired normal and tumor PDO (#175), analyzed at four days. Results are shown as the fold-change respect to the untreated organoids ± SD of triplicates of an experiment repeated twice. *P* values were calculated using Student *t* test (two-sided). **E** Immunofluorescence analyses of the effect of the anti-CD98hc or anti-CD98hc-DM1 on a tumoral PDO. Organoids were treated with anti-CD98hc or anti-CD98hc-DM1 at 10 nM for 4 days and the effect on cell morphology was evaluated by inmunofluorecescence. CD98hc is stained in green, nuclei are stained in blue. Scale bars = 25 µm. Arrows indicate nuclear fragmentation

The potential relevance of CD98hc targeting in CRC using the anti-CD98hc ADC, was analyzed by carrying out functional studies on patient-derived organoids (PDOs). These studies were designed to examine not only the efficacy of the anti-CD98hc ADC on tumoral tissue, but also on PDOs from normal colonic tissue derived from the same patient. Western blotting studies of those paired organoids showed that CD98hc expression was higher in tumoral PDOs than in normal colon PDOs (Fig. 5A). CA2, used as a marker of normal colonic tissue [40], was in fact only detected in the organoids derived from the normal colon PDOs. Immunofluorescence studies showed that incubation of tumoral PDOs with the anti-CD98hc^ECTO^ antibody for 1.5 h resulted in accumulation of the antibody at the cell surface (Fig. 5B). This staining was observed in the outermost surface layer of cells and was more difficult to detect in the cells located internally in the PDO. As expected from limited oxygen and nutrients and differentiation, nuclear fragmentation indicative of apoptosis was observed in cells lining the central lumen of the PDOs.

To explore the antiproliferative effect of the anti-CD98hc-DM1 ADC, three tumoral PDO cultures were treated with 10 nM of the anti-CD98hc-DM1 ADC for 4 days. Treatment with the anti-CD98hc-DM1 antibody decreased cell viability in all cases (Fig. 5C). The effect of the anti-CD98hc-DM1 ADC was found to be dose-dependent (Fig. 5D). Moreover, when the action of the anti-CD98hc-DM1 ADC was explored in paired normal and tumoral PDOs, a substantially higher inhibitory effect was observed in the tumoral samples (Fig. 5D). Immunofluorescence experiments showed that the anti-CD98hc-DM1 ADC caused alteration of the normal structure of the cells as well nuclear fragmentation, characteristic of apoptotic cell death (arrows in Fig. 5E). In contrast, treatment with the nude anti-CD98hc antibody did not induce any substantial change in cell viability or morphological alterations of the PDOs (Fig. 5E).

### Antitumoral in vivo action of the CD98hc-DM1 ADC

**Fig. 6:**
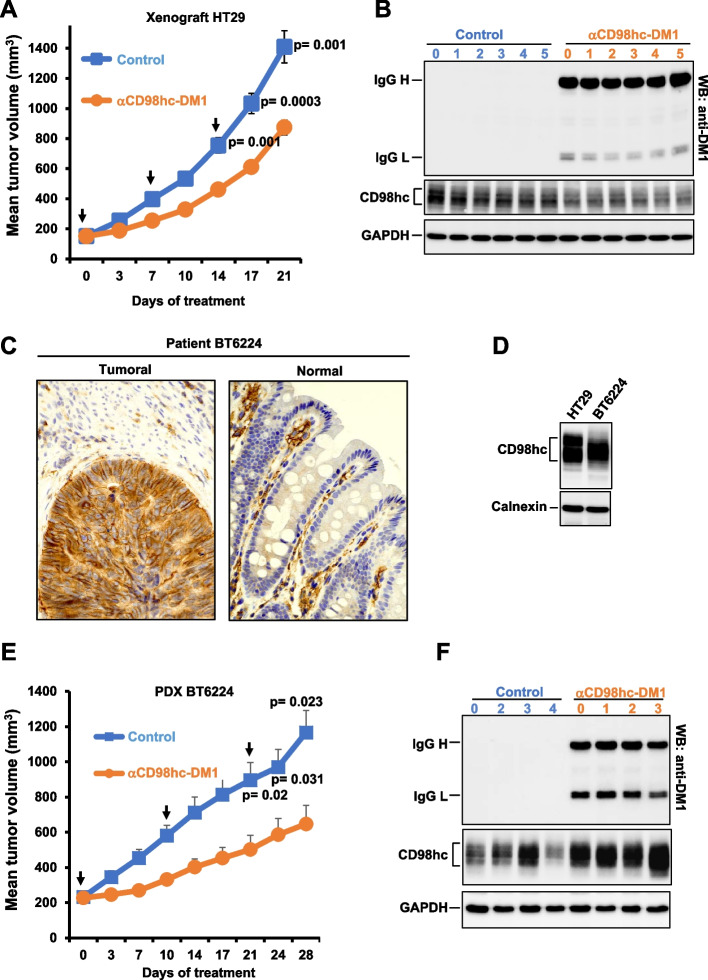
The anti-CD98hc-DM1 ADC has antitumoral activity on in vivo CRC models. **A** Evaluation of the antitumoral effect of anti-CD98hc-DM1 on tumor growth in nude mice implanted with HT29 cells. Arrows indicate days of administration of anti-CD98hc-DM1 (15 mg/Kg). Data are plotted as mean tumor volumes ± SEM. *P* values were calculated using Student’s *t* test (two-sided). **B** Expression of anti-CD98hc-DM1 and CD98hc in the tumors from mice. Tumor samples were obtained on day 21 after initiation of treatments (seven days after the last treatment). Tissue extracts of the tumors were used to analyze the levels of expression of anti-CD98hc-DM1 and CD98hc by Western blot with anti-DM1 and anti-CD98hc^V509^ antibodies, respectively. GAPDH was used as a loading control. **C** Immunohistochemical staining of CD98hc with the anti-CD98hc^V509^ antibody in normal and tumoral tissue of patient BT6224 used to generate the PDX. Magnification: 40 X. **D** Analysis of expression of CD98hc by Western blot with the anti-CD98hc^V509^ antibody in a PDX derived from BT6224, and in HT29 cells. Calnexin was used as a loading control. **E** Effect of anti CD98hc-DM1 on tumor growth in nude mice carrying BT6224-derived PDXs. Arrows indicate days of administration of anti-CD98hc-DM1 (15 mg/Kg). Data are plotted as mean tumor volumes ± SEM. *P* values were calculated using Student *t* test (two-sided). **F** Evaluation of anti-CD98hc-DM1 and CD98hc in tumoral samples by western blot with anti-DM1 and anti-CD98hc^V509^ antibodies, respectively. GAPDH was used as a loading control

Next, the effect of the anti-CD98hc-DM1 ADC was investigated in two in vivo models of colon cancer, one derived from HT29 cells xenografted in nude mice, and the other derived from tissue implanted from a patient with CRC which was established as a PDX. For the first model, mice were injected subcutaneously with HT29 cells into the right flank in their back and when tumors reached a mean volume of 150 mm^3^, the animals were randomized to receive vehicle (*n* = 6) or anti-CD98hc-DM1 (*n* = 6) intraperitoneally every week (three doses in total). Treatment with anti-CD98hc-DM1 produced a significant reduction in tumor growth in that in vivo model (Fig. 6A) and favored survival (supplementary Fig. 4A). Body weights of the animals in the treated and control groups did not show substantial changes throughout the experiment (supplementary Fig. 4B). In contrast, treatment of mice with the nude anti-CD98hc antibody or the free drug DM1 did not influence tumor growth or survival (supplementary Fig. 4C and D).

Tumors from control or anti-CD98hc-DM1-treated mice were removed at the end of the experiment (one week after the last treatment). Western blotting with the anti-DM1 antibody showed that the tumors of the treated mice accumulated anti-CD98hc-DM1, even one week after the last treatment (Fig. 6B and supplementary Fig. 4F). These western studies also showed that the tumors expressed CD98hc. Additional western studies showed significant up-regulation of pHistone-H3 in mice treated with anti-CD98hc-DM1 (supplementary Fig. 4E and F). In contrast to the in vitro studies, caspase 3 and PARP cleavage was not observed in the samples treated with anti-CD98hc-DM1. This may be due to different experimental settings.

For the second model, mice were inoculated through a subcutaneous incision with pieces of a tumor (BT6224) from a patient with colon cancer. Immunostaining of normal and tumoral surgical samples from this patient, showed substantial expression of CD98hc in the tumor, while in normal villi CD98hc staining was limited to the inflammatory stromal component (Fig. 6C). This tumor was propagated in nude mice. Western blotting analysis of tumors resected from these mice showed that the level of CD98hc was analogous to that of in vitro cultured HT29 cells (Fig. 6D). Moreover, immunohistochemical analyses of tissue derived from these PDXs showed that CD98hc was expressed at the cell membrane of the resected tumors (supplementary Fig. 4G). Treatment with anti-CD98hc-DM1 of mice carrying established BT6224 PDXs resulted in a significant reduction of growth (Fig. 6E) and potentiated survival (supplementary Fig. 4H). That reduction of tumor size was already evident after one week of treatment with the ADC, and the differences in tumor size were maintained along the duration of the experiment. Again, as occurred with the xenografted cell line in vivo model, the body weights of the animals in the treated and control groups did not show substantial changes or differences (supplementary Fig. 4I), and the tumors accumulated anti-CD98hc-DM1 (Fig. 6F and supplementary Fig. 4J).

## Discussion

The development of novel strategies for the therapy of neoplastic diseases is a medical need, and CRC is not an exception since progression of the disease due to failure of available antitumoral agents renders CRC incurable. To increase the therapeutic options in cancer, an approach that is being used is the identification of cell surface proteins preferentially expressed in tumors, with the purpose of developing tools, either chemical of cellular, to fight the disease [11]. Identification of these cell surface targets may not only be useful for a type of tumor, but also for others in which the same protein may be overexpressed or differentially expressed with respect to normal tissues. Following that idea, we formerly launched a study of the surfaceome of triple negative breast tumors. In that work, mass-spectrometric as well as genomic analyses led to the identification of CD98hc as one of the most up-regulated proteins in those tumors as compared to normal breast tissue [14]. That finding represented the basis for the development of a study in which CD98hc was validated as a potential ADC target in the above-mentioned type of breast cancer. These precedents raised the possibility that CD98hc could also be overexpressed in other tumoral types. Given the need for novel therapeutic strategies in advanced CRC, the possibility that CD98hc could represent a novel actionable target was the idea behind the studies herewith described.

A first step taken was to explore whether CD98hc was expressed in colon cancer and if its expression resulted higher than in normal colon tissue. To that end, we analyzed CD98hc expression in a cohort of 27 patients from which paired samples were available. Quantitative western blotting studies demonstrated that tumoral tissues expressed higher amounts of CD98hc than the paired normal counterparts. This result was further confirmed by immunohistochemical experiments, which also showed that CD98hc is a cell surface protein preferentially expressed by tumor cells. It should be noted that while normal intestinal epithelia did not show staining in those experiments, western blotting analyses identified expression of CD98hc in normal tissue samples. The explanation for such likely relies in the fact that CD98hc was expressed in the stromal compartment of the villi, as shown by the immunohistochemical studies. These results are in line with recently published works [33, 34] which demonstrated lack of CD98hc immunohistochemical staining in normal colonic mucosa and various benign lesions. Finally, revision of online available data from several sources confirmed that CD98hc expression was significantly higher in colorectal tumoral tissues when compared to normal ones. In conclusion, our results and those of others demonstrated that CD98hc expression is augmented in CRC.

Together with the precedents of the therapeutic effectiveness of an ADC against CD98hc in triple negative breast cancer, the finding CD98hc was differentially expressed in CRC when compared to normal colon tissue, represented an attractive setting in which to explore whether an anti-CD98hc ADC could be efficacious in colon cancer. To that end, several analyses were performed using different cell models, including cell lines, PDOs and PDXs. All the analyzed models expressed CD98hc. Furthermore, immunofluorescence studies carried out with an antibody that recognizes native anti-CD98hc showed that the anti-CD98hc-CD98hc complex internalized in colon cancer cells and was targeted to the lysosomal compartment. These studies are relevant as they confirmed (i) expression, (ii) internalization and (iii) trafficking of CD98hc to lysosomes in colon cancer cells, three prerequisites necessary for the successful targeting of a cell surface protein with an ADC.

The results reported here show that an ADC against CD98hc provoked a strong antiproliferative effect in all five colon cancer cell lines tested. The specificity of the anti-CD98hc-DM1 ADC was demonstrated using two different genetic strategies based on the decrease of CD98hc by shRNA or its deletion by CRISPR/Cas9. In fact, downregulation or deletion of CD98hc compromised the antitumoral activity of the anti-CD98hc-DM1 ADC. Of note, none of the two strategies aimed at reducing the levels of CD98hc significantly affected cell proliferation, suggesting that the function of CD98hc is largely dispensable in the models studied. This finding is in line with a previous study [51], which generated a colon adenocarcinoma cell line LS174T lacking CD98hc that grew like the parental cell line. However, other reports have indicated that decreasing CD98hc inhibits proliferation [30]. Therefore, the relevance of CD98hc in proliferation may depend on the cellular context.

The knockout and knockdown data obtained in HT29 cells together with data from non-tumoral cells, indicate that expression of CD98hc may represent a relevant factor in the antiproliferative action of the anti-CD98hc-DM1 ADC. Tissues with low expression of CD98hc may be spared from the action of agents targeting CD98hc. However, the ADC could exert undesired toxicity on normal tissues expressing high levels of CD98. In this respect it is interesting to note that tumoral tissues usually contain higher levels of the protein than the normal counterparts, as supported by the in silico studies. This fact opens the possibility of using agents targeting CD98hc in tumors in which expression of the protein is elevated.

Mechanistic studies showed that the anti-CD98hc-DM1 caused mitotic arrest that was followed by cell death caused by mitotic catastrophe. Mitotic arrest was suggested by the increased levels of pBUBR1 and pH3, together with increased levels of cyclin A and B. The latter cyclins are able to interact with CDK1, promoting entry in mitosis, and their degradation is required for mitotic exit [52]. Such mechanism of action is like that reported for other ADCs carrying the same payload [53]. In contrast, the nude anti-CD98hc antibody did not significantly affect the proliferation of colon cancer cell lines, indicating that the payload coupled to the antibody is responsible for the arrest in cell proliferation. It is relevant to indicate that a recent report has described antitumoral properties of nude anti-CD98hc antibodies in multiple myeloma [15]. The nude antibody that we used against CD98hc not only failed to affect proliferation of CRC cells but also of triple negative breast cancer cells [14]. These differences may rely in the effectiveness of targeting CD98hc in different tissues or simply because the use of distinct antibodies.

Especially relevant were the studies carried out in the two in vivo models of colon cancer as well as the PDOs. In the in vivo models, a limited number of treatments with the CD98hc-directed ADC caused a statistically significant inhibition of tumor growth. Interestingly, biochemical inspection of the ADC in the tumor samples by Western blotting revealed that it accumulated in the tumoral tissues of the treated mice even one week after their last treatment. This is relevant as it indicates that the ADC targets the tumoral cells in a physiological context. Importantly, the anti-CD98hc-DM1 did not induce signs of toxicity in either of the two in vivo models. However, this lack of toxicity must be interpreted with caution, as the anti-CD98hc antibody used to generate the ADC recognizes human but not murine CD98hc. In fact, that situation impedes fair assessment of the potential toxicities of the anti-CD98hc-ADC which could derive from specific action on endogenous murine CD98hc. Studies using immune-competent mouse models injected with mouse colon cancer cell lines and treated with a CD98hc-directed ADC able to recognize the murine protein will help in evaluating the toxicity and efficacy of the ADC in a more complete physiological context. Yet, while this represents a limitation of our study, some data suggest that ADCs against CD98hc could be used therapeutically despite its expression in an ample number of normal tissues [14, 15]. On one hand, the experiments with the PDOs showed that a therapeutic window exists with respect to the antiproliferative action of the anti-CD98hc-DM1. In fact, its antiproliferative action on normal organoids was lower as compared to the action on tumoral ones. On the other hand, an anti-CD98hc antibody with antitumor capabilities that recognizes glycoforms of CD98hc exclusively present in multiple myeloma cells has been recently generated [15]. As mentioned above, such antibody exerted antitumoral action on myeloma cells.

In summary, here we show that CD98hc is overexpressed in colon cancer and can be targeted with therapeutic purposes. The evaluation of anti-CD98hc ADCs in the clinic is needed to inform about feasibility of CD98hc targeting. Moreover, considering the increasing importance given to CD98hc as a potential therapeutic target in several types of tumors, it is expected that pharmacological alternatives to ADCs may be used to target this protein. Such alternatives include naked anti-CD98hc antibodies with demonstrated antitumor activity [54], antibodies targeting tumor specific CD98hc glycoforms [15], CAR-T derived strategies [55], aptamers [56] or chemicals that may decrease its function or levels [35]. Of note, agents that target the CD98hc partner protein LAT1 have demonstrated antitumor properties [57, 58].

## Conclusions

In this study we show that CD98hc is a cell surface protein differentially expressed in colon cancer with respect to normal colonic tissue. Cell biological as well as biochemical analyses demonstrated that CD98hc can efficiently internalize to the lysosomes. Considering that property, an antibody–drug conjugate that recognized CD98h was prepared. This modified antibody showed antitumoral activity in in vitro (cell lines and patient-derived organoids) and in vivo (xenografted CRC cells as well as patient-derived xenografts) models of CRC. These studies indicate that CD98hc may represent a novel ADC target in CRC.

### Supplementary Information

**Additional file 1: Supplementary Fig. 1**. A) Box plot showing *SLC3A2* gene expression levels in paired normal colon tissue and tumoral CRC tissue, using TNMplot online tool. FC = fold change. *P* value obtained using Mann Whitney U test. B) Box plot showing *SLC3A2* gene expression levels in normal colon tissue and tumoral CRC tissue samples. Data were obtained from the GEPIA2. *P* value obtained using one-way ANOVA test. C) Box plot showing *SLC3A2* gene expression levels in normal colon tissue and tumoral CRC tissue normal, using TNMplot online tool. *P* value obtained using Mann Whitney U test. D) Box plot showing the CD98hc score analyzed by immunohistochemistry in primary tumor, lymph node and liver metastases. *P* values were obtained using Student t test (two-sided). E) Immunohistochemical staining of CD98hc in representative samples from the study shown in D, assessed using with anti-CD98hc^V509^ antibody. Magnification: 40X. F) Box plot showing *SLC3A2* gene expression levels in tumor and metastatic tissue of CRC patients. T = tumor, *N* = normal and M = metastatic. *P* value obtained using Kruskal–Wallis test. G) Heatmap (top) and boxplot (bottom) representative of the expression of CD98hc in different normal and tumoral tissues, obtained from the TNMplot database. Tissues written in red represent significant differences by the Mann–Whitney test. H) Expression of CD98hc in different normal and tumoral tissues. Data obtained from the GENT2 database.

**Additional file 2: Supplementary Fig. 2.** A) Internalization of the anti-CD98hc^ECTO^ antibody in SW480 cells, analyzed by immunofluorescence. Scale bar = 25 μm. The cells were seeded on coverslips and treated with 10 nM of anti-CD98hc^ECTO^ for the times indicated. The images at the right correspond to magnifications of a cell present in the images obtained at 24 h. The colocalization of CD98hc and LAPM1 is show in the merged images. B) Preparation of the antibody–drug conjugate targeting CD98hc. The coupling of DM1 to the anti-CD98hc^ECTO^ antibody was evaluated by Western blot using an anti-DM1 antibody. Twenty nanograms of anti-CD98hc^ECTO^-DM1, the nude anti-CD98hc^ECTO^, trastuzumab or T-DM1 were used to detect DM1 (upper panel) and the total amount of protein was evaluated by stain-free blot (lower image). Trastuzumab and T-DM1 were used as a negative and positive controls. C-E) FACS analyses in HT29 (C), cells dissociated from patient PDX BT6224 (D) and human tumoral organoid #48 (E) using anti-CD98hc-DM1 as primary antibody. The red histogram correspond to signals from cells incubated with the secondary antibody alone, whereas the blue histograms represent the fluorescence due to the expression of CD98hc.

**Additional file 3: Supplementary Fig. 3.** A) Dose–response analyses of the effect of anti-CD98hc-DM1 on the proliferation of parental and CD98hc CRISPR #5 and #11 HT29 cells. Cells were treated with anti-CD98hc-DM1 at the indicated doses for four days. Results are shown as the mean ± SD of quadruplicates of an experiment repeated three times. B) Expression of CD98hc in normal human fibroblasts (NHF) and immortalized keratinocytes (HaCaT), compared to CRC cell lines. Cell extracts (20 µg) were used to identify CD98hc by Western blot with the anti-CD98hc^V509^ antibody. Calnexin was used as a loading control. C) Dose–response analyses of the anti-CD98hc-DM1 ADC on NHF and HaCaT, compared to HT29 cells. Cells were treated with the ADC for four days at the indicated doses. The data are plotted as the percentage of MTT metabolization with respect to control. Results are shown as the mean ± SD of quadruplicates of an experiment repeated two times. D) Evaluation of the effect of anti-CD98hc-DM1 (10 nM, 48 h) on the distribution of the different cell cycle phases in HT29 and HCT116 cell lines. E) Immunofluorecescence analyses of the action of anti-CD98hc-DM1 on spindle assembly and organization on HCT116 cells treated with CD98hc-DM1 (10 nM) for 48 h. β-Tubulin (green), DAPI (blue). Scale bars = 7.5 µm. F). Detection of giant multinucleated cells or altered nuclear structures after anti-CD98hc-DM1 treatment. HCT116 and HT29 cells were treated with 10 nM anti-CD98hc^ECTO^-DM1 for 72 h, fixed and stained for nucleoporin p62 (red) and DNA (blue). Scale bar = 10 μm. The arrows indicate giant multinucleated cells.

**Additional file 4: Supplementary Fig. 4.** A) Kaplan–Meier survival curve of mice from the experiment performed in Fig. 6A. The Kaplan–Meier survival plot was created using a tumor volume threshold of 1,000 mm^3^. *P* values were calculated using one-sided log-rank tests. B) Effect of the anti-CD98hc ADC on the weight of mice xenografted with HT29 cells. Data are plotted as mean ± SD of six mice/group. C) Analysis of the antitumoral effect of naked anti-CD98hc and DM1 on tumor growth in nude mice implanted with HT29 cells. Arrows indicate days of administration of anti-CD98hc (15 mg/Kg) or DM1 (0.14 mg/Kg). Data are plotted as mean tumor volumes ± SEM. *P* values were calculated using Student’s *t* test (two-sided). D) Kaplan–Meier survival curve of mice from the experiment of panel C. The Kaplan–Meier survival plot was created using a tumor volume threshold of 650 mm^3^. *P* values were calculated using one-sided log-rank tests. E) Expression levels or phosphorylation of proteins involved in cell cycle and apoptosis in the tumors of the experiment performed in Fig. 6A. Tumor samples were obtained on day 21 after initiation of treatments (seven days after the last treatment). Tissue extracts of the tumors were used to analyze the levels of expression of pH3, PARP, pH2AX, pCDK1 and cleaved Caspase 3. Stain free blot was analyzed to verify equal loading. F) Quantitation of the levels of DM1 (data shown in Fig. 6B), pH3, PARP, pH2AX, pCDK1 and cleaved Caspase 3 of the experiments shown in panel E. The graphs represent the mean intensity (arbitrary units) ± SD of the different proteins present in control (C) or treated (anti-CD98hc-DM1) mice groups. *P* values were calculated using Student’s *t* test (two-sided). G) Immunohistochemical detection of CD98hc in PDX BT6224 (P2M1: passage 2, mouse #1) using the anti-CD98hc^V509^ antibody. H) Kaplan–Meier survival curve of mice from the experiment performed in Fig. 6E. The Kaplan–Meier survival plot was created using a tumor volume threshold of 800 mm^3^. *P* values were calculated using one-sided log-rank tests. I) Effect of the anti-CD98hc ADC on the weight of mice xenografted with PDX BT6224. Data are plotted as mean ± SD of four mice/group. J) Quantitation of the levels of DM1 of the experiments performed in Fig. 6F. The graphs represent the mean intensity (arbitrary units) ± SD of IgG Heavy and Light chains coupled to DM1 present in control (C) or treated (anti-CD98hc-DM1) groups. *P* values were calculated using Student’s *t* test (two-sided).

## Data Availability

Data available from authors; materials depending on their availability (particularly in the case of patient samples).

## Abbreviations

CRC: Colorectal cancer
ADCs: Antibody–drug conjugates
DMEM: Dulbecco’s modified Eagle medium
FBS: Fetal bovine serum
T-DM1: Trastuzumab-emtansine
MTT: 3-(4, 5-Dimethylthiazol-2-yl)-2, 5-Diphenyltetrazolium Bromide
BSA: Bovine serum albumin
PDXs: Patient Derived Xenografts
PDOs: Patient-derived organoids
