# Supplementary figures and images for: An amino acid transporter subunit as an antibody–drug conjugate target in colorectal cancer
Source: J Exp Clin Cancer Res. 2023 Aug 9;42:200. doi: 10.1186/s13046-023-02784-0 (PMC10410906; doi:10.1186/s13046-023-02784-0)

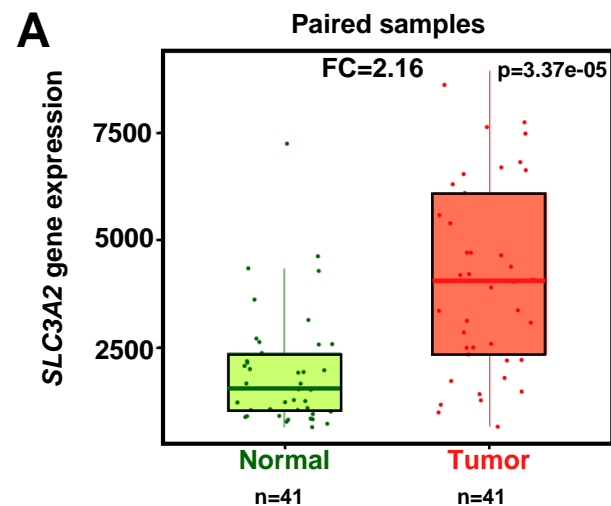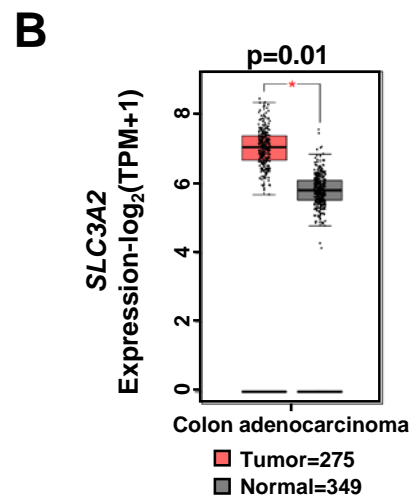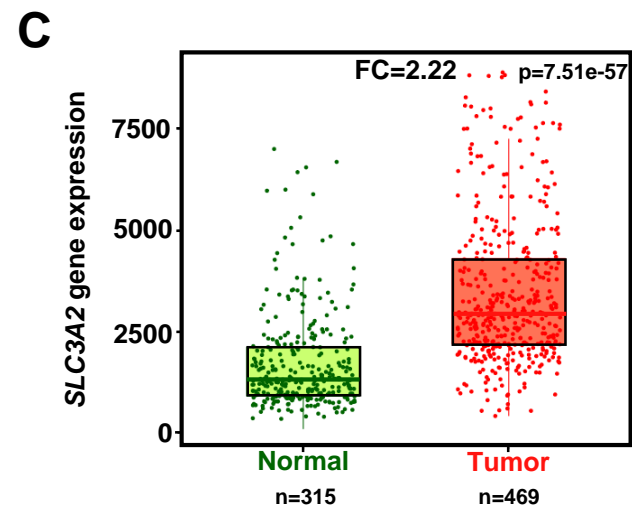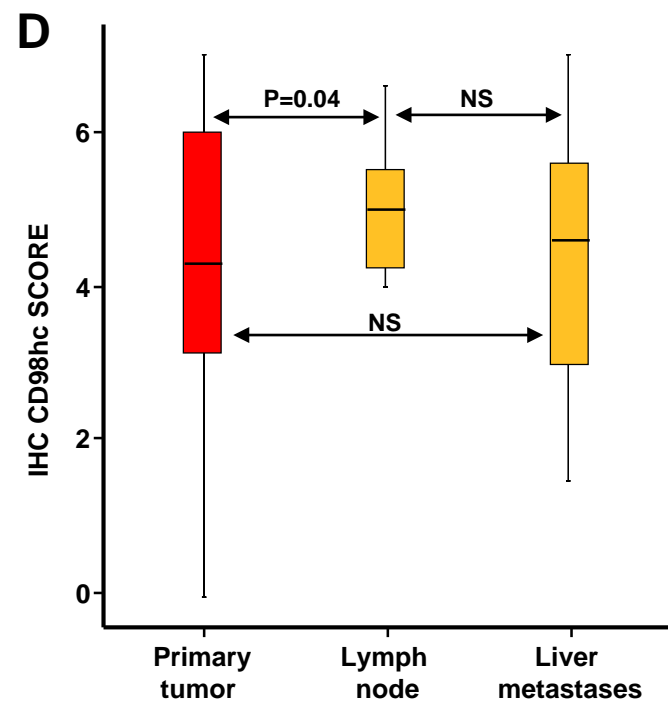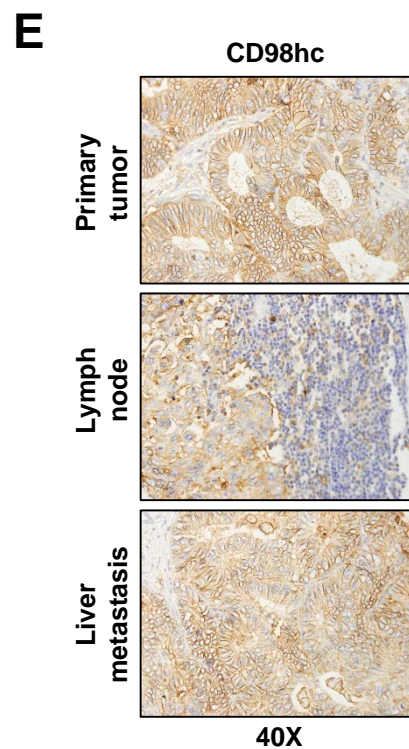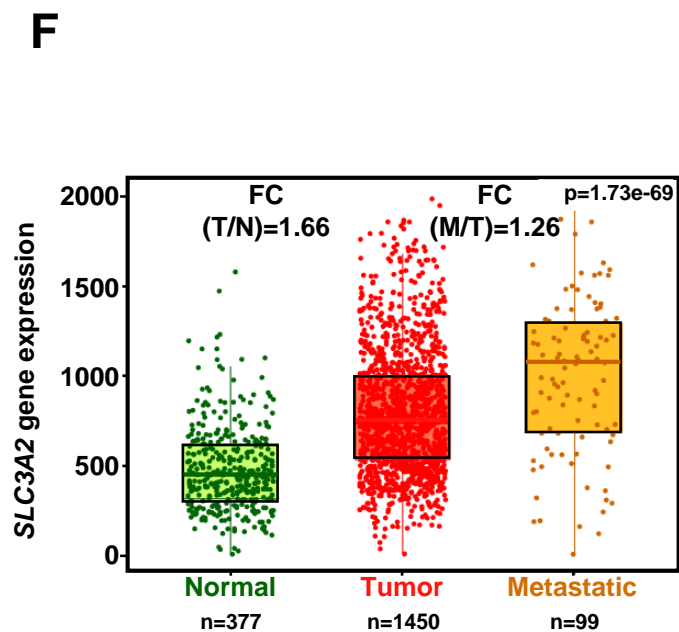

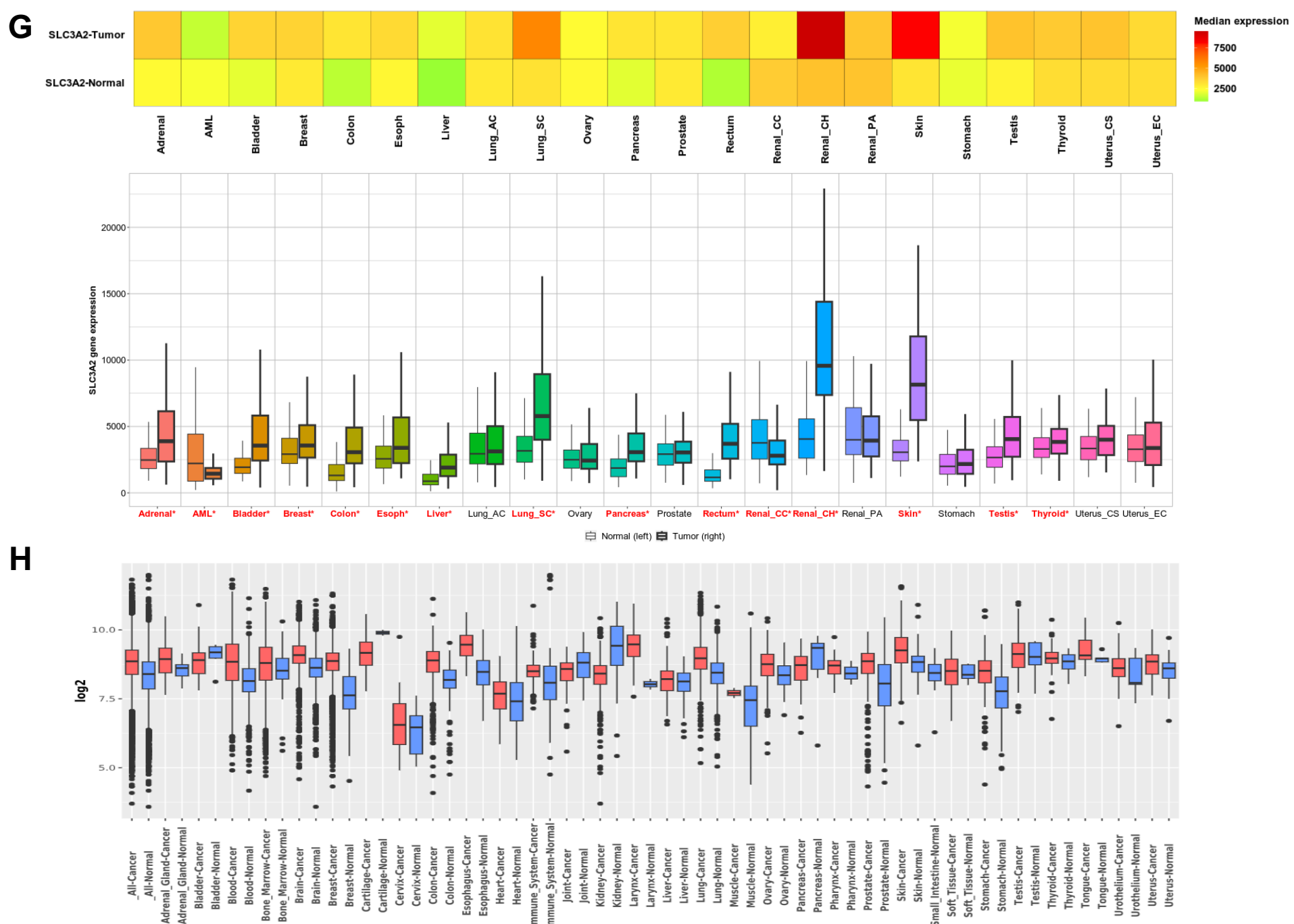

Supplementary Figure 1

Supplement: Supplementary file 1 — Additional file 1: Supplementary Fig. 1. A) Box plot showing SLC3A2 gene expression levels in paired normal colon tissue and tumoral CRC tissue, using TNMplot online tool. FC = fold change. P value obtained using Mann Whitney U test. B) Box plot showing SLC3A2 gene expression levels in normal colon tissue and tumoral CRC tissue samples. Data were obtained from the GEPIA2. P value obtained using one-way ANOVA test. C) Box plot showing SLC3A2 gene expression levels in normal colon tissue and tumoral CRC tissue normal, using TNMplot online tool. P value obtained using Mann Whitney U test. D) Box plot showing the CD98hc score analyzed by immunohistochemistry in primary tumor, lymph node and liver metastases. P values were obtained using Student t test (two-sided). E) Immunohistochemical staining of CD98hc in representative samples from the study shown in D, assessed using with anti-CD98hcV509 antibody. Magnification: 40X. F) Box plot showing SLC3A2 gene expression levels in tumor and metastatic tissue of CRC patients. T = tumor, N = normal and M = metastatic. P value obtained using Kruskal–Wallis test. G) Heatmap (top) and boxplot (bottom) representative of the expression of CD98hc in different normal and tumoral tissues, obtained from the TNMplot database. Tissues written in red represent significant differences by the Mann–Whitney test. H) Expression of CD98hc in different normal and tumoral tissues. Data obtained from the GENT2 database. [file 13046_2023_2784_MOESM1_ESM.pdf]

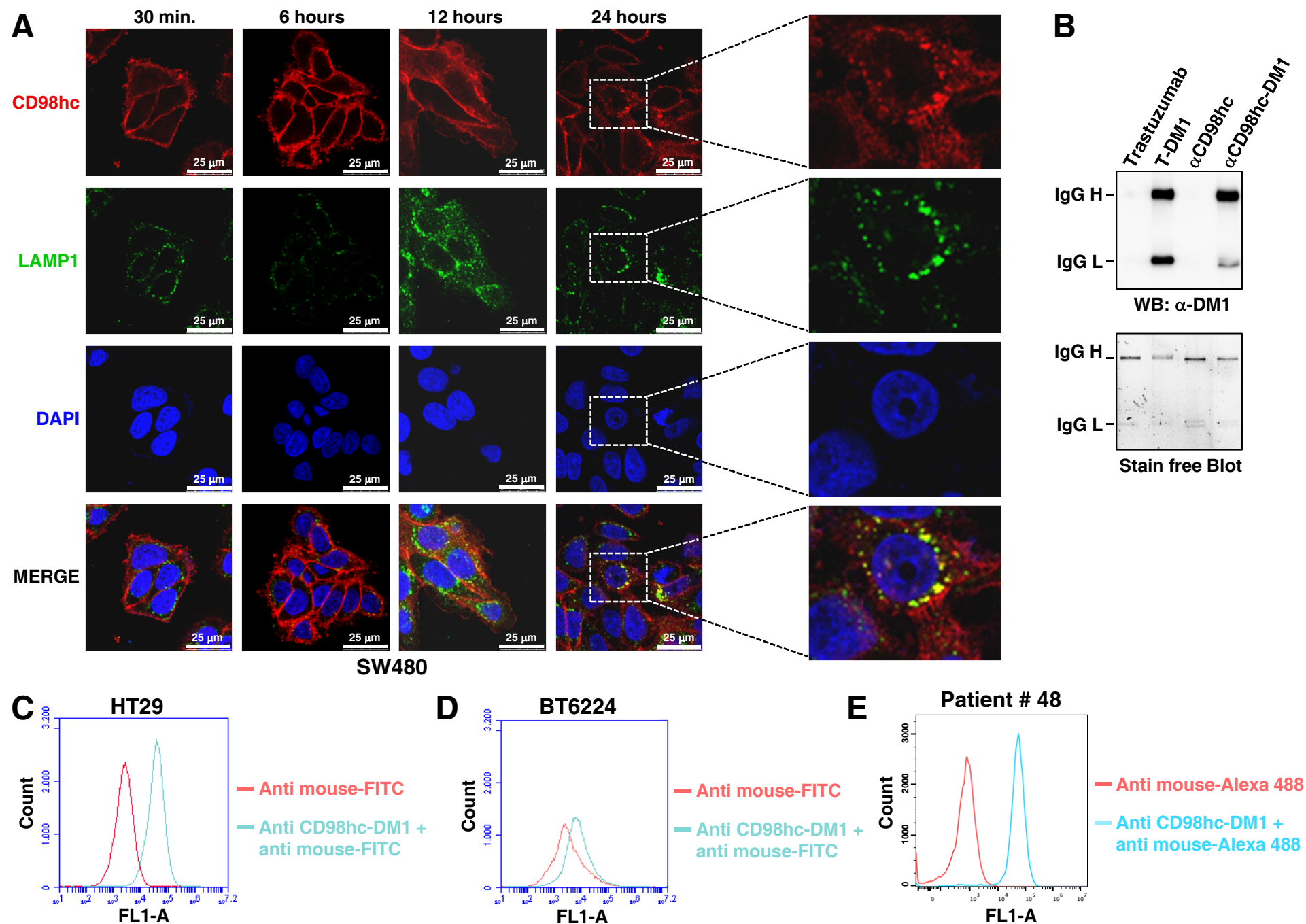

Supplementary Figure 2

Supplement: Supplementary file 2 — Additional file 2: Supplementary Fig. 2. A) Internalization of the anti-CD98hcECTO antibody in SW480 cells, analyzed by immunofluorescence. Scale bar = 25 μm. The cells were seeded on coverslips and treated with 10 nM of anti-CD98hcECTO for the times indicated. The images at the right correspond to magnifications of a cell present in the images obtained at 24 h. The colocalization of CD98hc and LAPM1 is show in the merged images. B) Preparation of the antibody–drug conjugate targeting CD98hc. The coupling of DM1 to the anti-CD98hcECTO antibody was evaluated by Western blot using an anti-DM1 antibody. Twenty nanograms of anti-CD98hcECTO-DM1, the nude anti-CD98hcECTO, trastuzumab or T-DM1 were used to detect DM1 (upper panel) and the total amount of protein was evaluated by stain-free blot (lower image). Trastuzumab and T-DM1 were used as a negative and positive controls. C-E) FACS analyses in HT29 (C), cells dissociated from patient PDX BT6224 (D) and human tumoral organoid #48 (E) using anti-CD98hc-DM1 as primary antibody. The red histogram correspond to signals from cells incubated with the secondary antibody alone, whereas the blue histograms represent the fluorescence due to the expression of CD98hc. [file 13046_2023_2784_MOESM2_ESM.pdf]

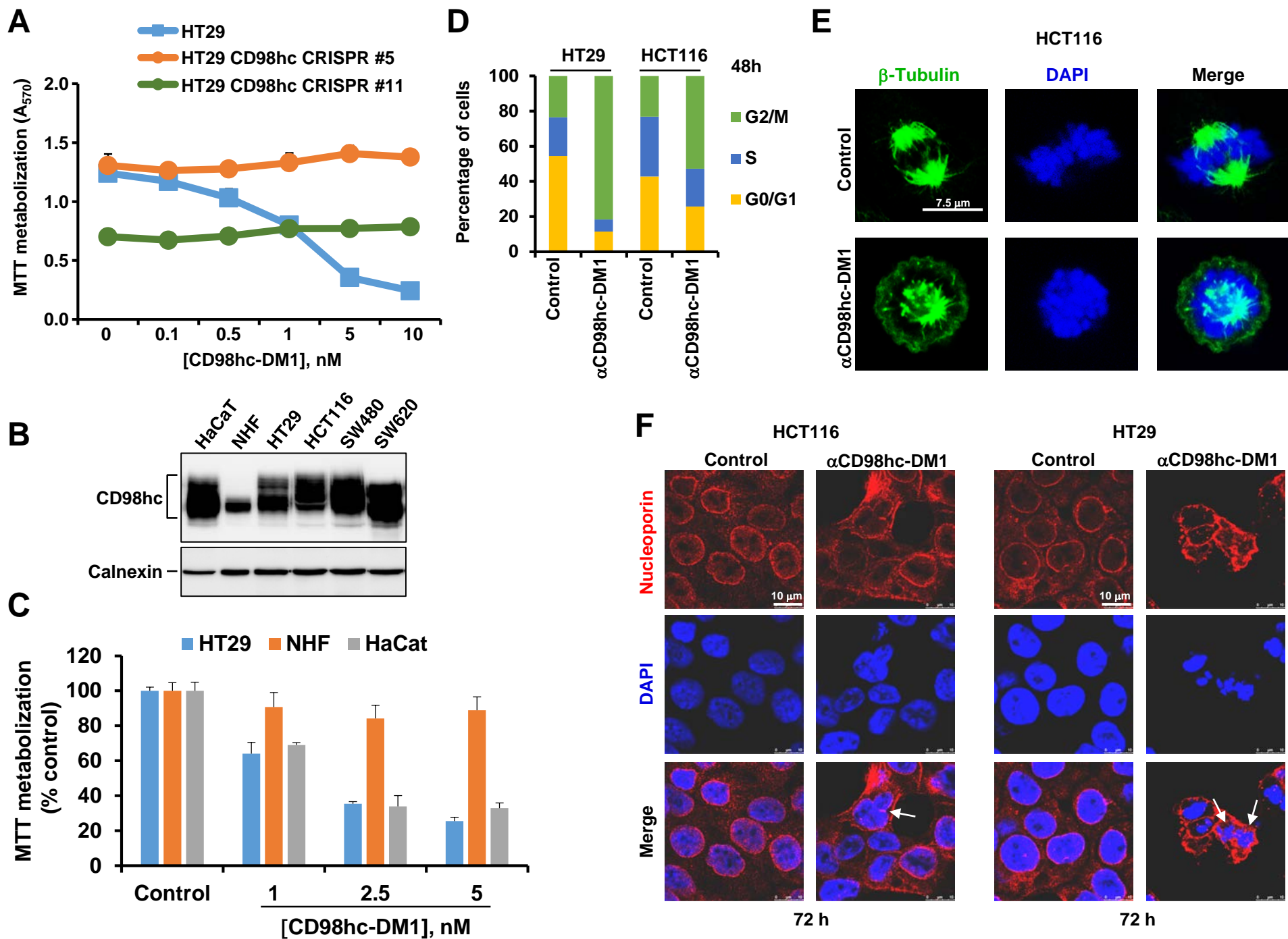

Supplementary Figure 3

Supplement: Supplementary file 3 — Additional file 3: Supplementary Fig. 3. A) Dose–response analyses of the effect of anti-CD98hc-DM1 on the proliferation of parental and CD98hc CRISPR #5 and #11 HT29 cells. Cells were treated with anti-CD98hc-DM1 at the indicated doses for four days. Results are shown as the mean ± SD of quadruplicates of an experiment repeated three times. B) Expression of CD98hc in normal human fibroblasts (NHF) and immortalized keratinocytes (HaCaT), compared to CRC cell lines. Cell extracts (20 µg) were used to identify CD98hc by Western blot with the anti-CD98hcV509 antibody. Calnexin was used as a loading control. C) Dose–response analyses of the anti-CD98hc-DM1 ADC on NHF and HaCaT, compared to HT29 cells. Cells were treated with the ADC for four days at the indicated doses. The data are plotted as the percentage of MTT metabolization with respect to control. Results are shown as the mean ± SD of quadruplicates of an experiment repeated two times. D) Evaluation of the effect of anti-CD98hc-DM1 (10 nM, 48 h) on the distribution of the different cell cycle phases in HT29 and HCT116 cell lines. E) Immunofluorecescence analyses of the action of anti-CD98hc-DM1 on spindle assembly and organization on HCT116 cells treated with CD98hc-DM1 (10 nM) for 48 h. β-Tubulin (green), DAPI (blue). Scale bars = 7.5 µm. F). Detection of giant multinucleated cells or altered nuclear structures after anti-CD98hc-DM1 treatment. HCT116 and HT29 cells were treated with 10 nM anti-CD98hcECTO-DM1 for 72 h, fixed and stained for nucleoporin p62 (red) and DNA (blue). Scale bar = 10 μm. The arrows indicate giant multinucleated cells. [file 13046_2023_2784_MOESM3_ESM.pdf]

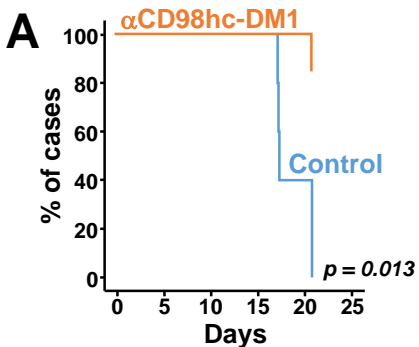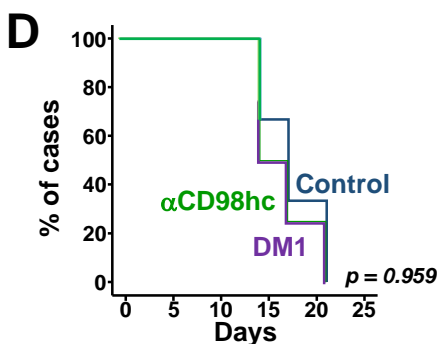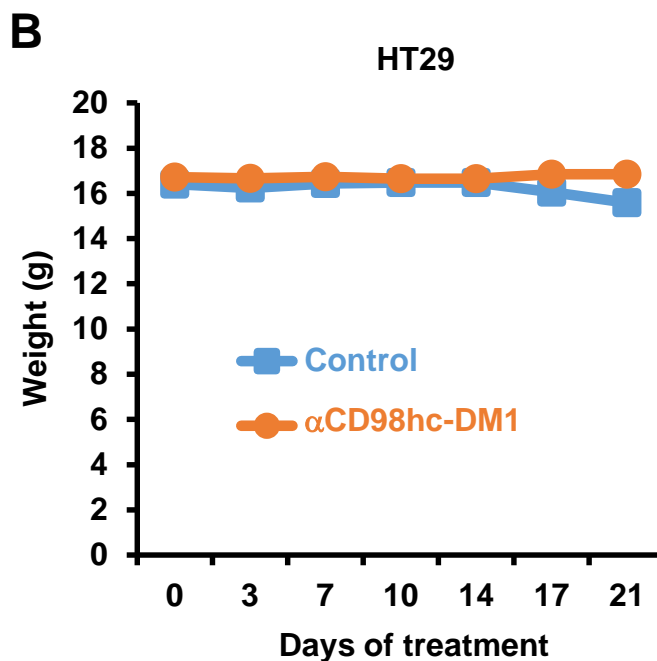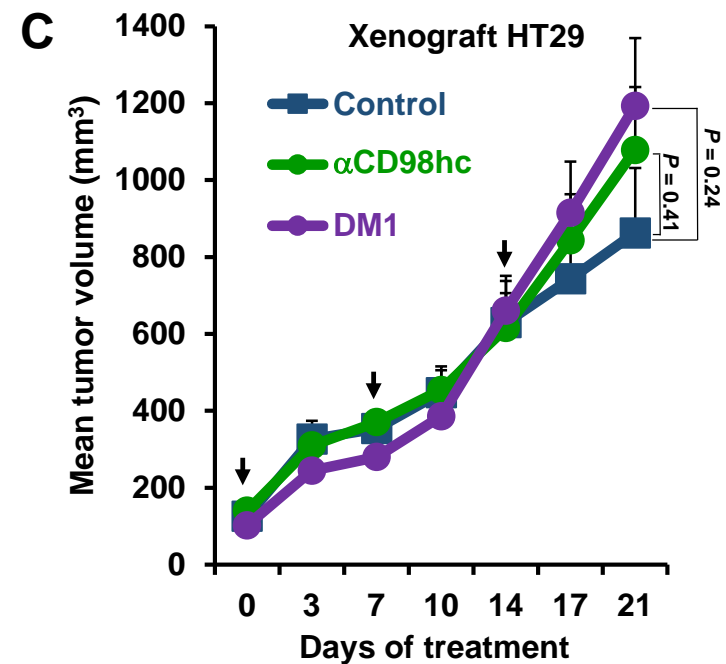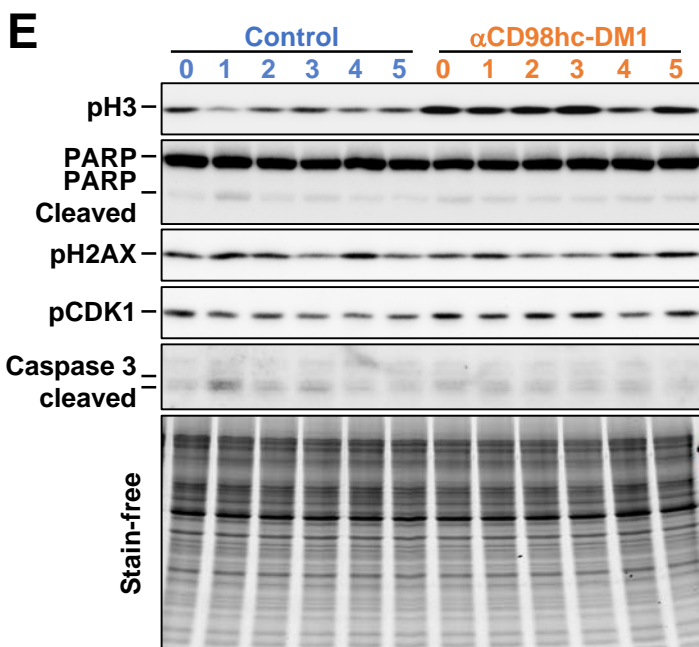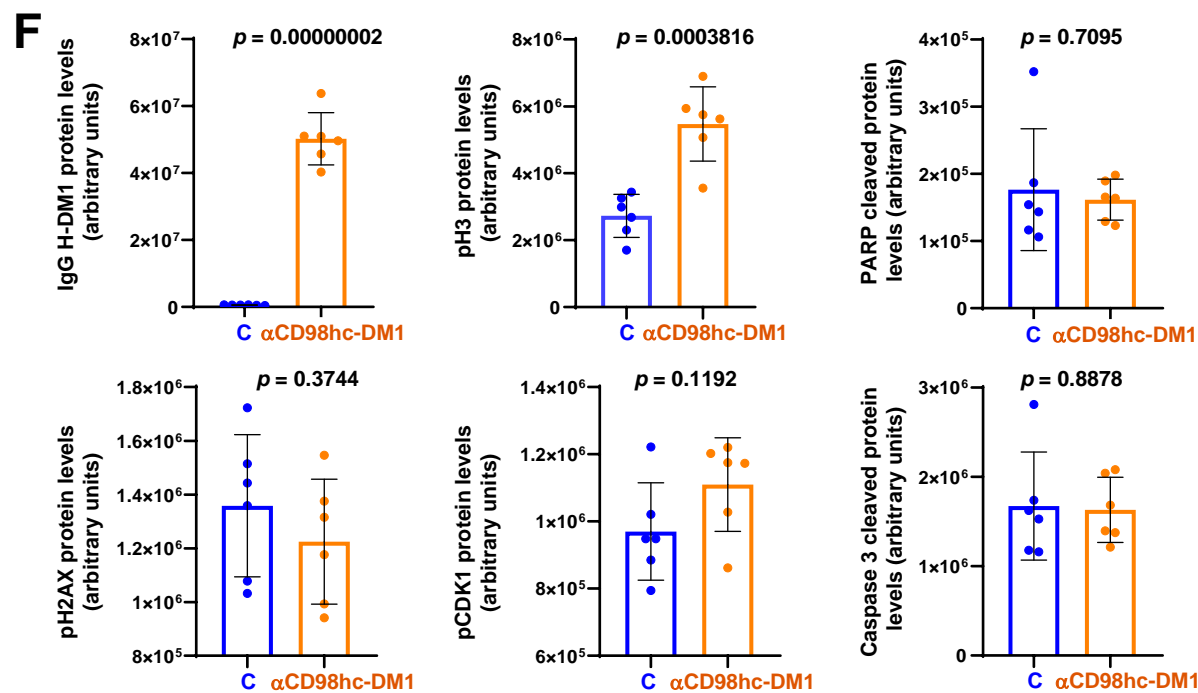

**G****PDX BT6224 (P2M1)**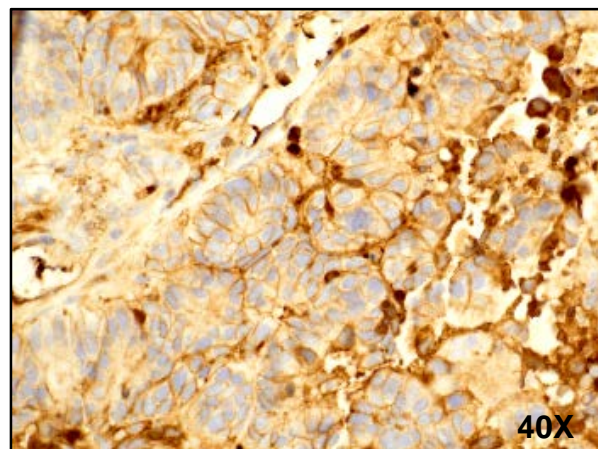**H****PDX BT6224**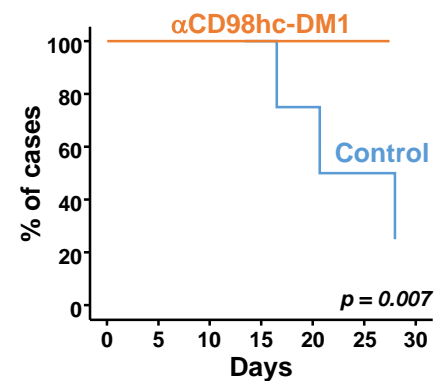**I****PDX BT6224**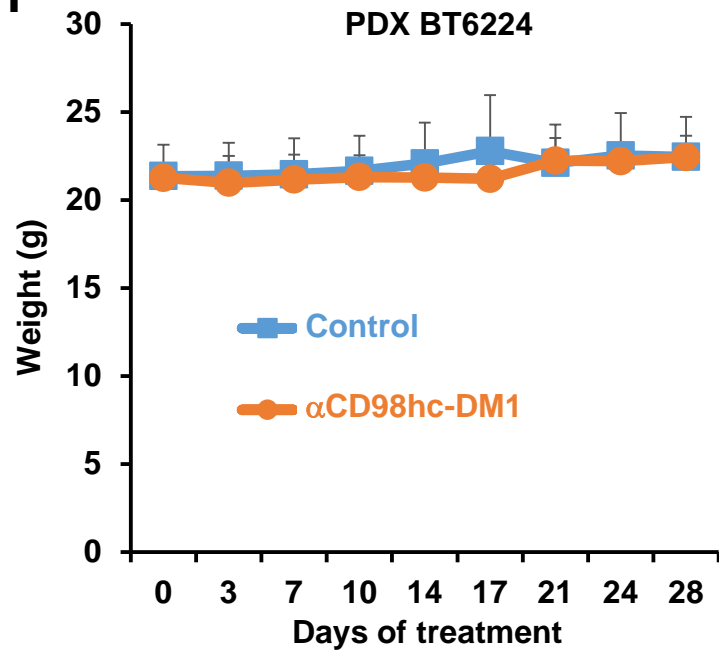**J****PDX BT6224**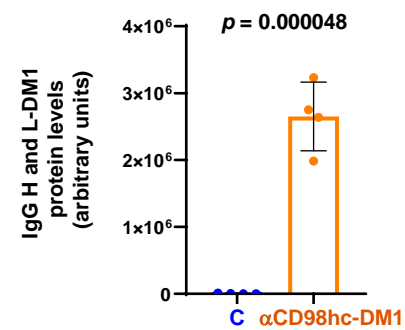**Supplementary Figure 4**

Supplement: Supplementary file 4 — Additional file 4: Supplementary Fig. 4. A) Kaplan–Meier survival curve of mice from the experiment performed in Fig. 6A. The Kaplan–Meier survival plot was created using a tumor volume threshold of 1,000 mm3. P values were calculated using one-sided log-rank tests. B) Effect of the anti-CD98hc ADC on the weight of mice xenografted with HT29 cells. Data are plotted as mean ± SD of six mice/group. C) Analysis of the antitumoral effect of naked anti-CD98hc and DM1 on tumor growth in nude mice implanted with HT29 cells. Arrows indicate days of administration of anti-CD98hc (15 mg/Kg) or DM1 (0.14 mg/Kg). Data are plotted as mean tumor volumes ± SEM. P values were calculated using Student’s t test (two-sided). D) Kaplan–Meier survival curve of mice from the experiment of panel C. The Kaplan–Meier survival plot was created using a tumor volume threshold of 650 mm3. P values were calculated using one-sided log-rank tests. E) Expression levels or phosphorylation of proteins involved in cell cycle and apoptosis in the tumors of the experiment performed in Fig. 6A. Tumor samples were obtained on day 21 after initiation of treatments (seven days after the last treatment). Tissue extracts of the tumors were used to analyze the levels of expression of pH3, PARP, pH2AX, pCDK1 and cleaved Caspase 3. Stain free blot was analyzed to verify equal loading. F) Quantitation of the levels of DM1 (data shown in Fig. 6B), pH3, PARP, pH2AX, pCDK1 and cleaved Caspase 3 of the experiments shown in panel E. The graphs represent the mean intensity (arbitrary units) ± SD of the different proteins present in control (C) or treated (anti-CD98hc-DM1) mice groups. P values were calculated using Student’s t test (two-sided). G) Immunohistochemical detection of CD98hc in PDX BT6224 (P2M1: passage 2, mouse #1) using the anti-CD98hcV509 antibody. H) Kaplan–Meier survival curve of mice from the experiment performed in Fig. 6E. The Kaplan–Meier survival plot was created using a tumor volume threshold of [file 13046_2023_2784_MOESM4_ESM.pdf]
